# Gender Differences in Quality of Life and Psycho‐Oncological Needs During the First Year After Melanoma Diagnosis

**DOI:** 10.1002/pon.70335

**Published:** 2025-11-19

**Authors:** Susanne Dugas‐Breit, Jessica Hassel, Martin Dugas, Hans‐Joachim Schulze

**Affiliations:** ^1^ Medical Faculty Heidelberg Department of Dermatology and National Center for Tumor Diseases (NCT) NCT Heidelberg Heidelberg University a Partnership Between DKFZ and University Hospital Heidelberg Heidelberg Germany; ^2^ Institute of Medical Informatics Heidelberg University Hospital Heidelberg Germany; ^3^ Department of Dermatology Fachklinik Hornheide Münster Germany

**Keywords:** melanoma, psycho‐oncological care, quality of life, rehabilitation, sex differences

## Abstract

**Objective:**

This study investigated the course of general well‐being and health‐related quality of life (HRQoL) in working‐age melanoma patients during the first year following diagnosis. It also examines the use of psycho‐oncological counseling and rehabilitation, and their impact on QoL.

**Methods:**

Patients aged 18–65 years with stage 0 to IIIC melanoma were eligible for this single‐center, prospective cohort study. Following informed consent, clinical data and data on general well‐being (WHO‐5), HRQoL (FACT‐M) and need for psycho‐oncological care (Hornheider Screening Instrument) were collected at baseline and every three months over one year.

**Results:**

We included 221 melanoma patients (median age 51, range 19–65, 62% female). At baseline, 79% had melanoma stage IB or lower. After one year, 9% had progressed. 38% of patients showed a WHO‐5 score below 52% following diagnosis, regardless of tumor stage. Women with stage 0 to IIA melanoma had significantly lower HRQoL in the first six months than men (*p* = 0.010), and a higher need for psychological support (*p* < 0.001). There was considerable variability in QoL trajectories both within individuals (median variation 11%) and across patients. In general, 52% needed psycho‐oncological care at baseline, but neither counseling (24%) nor rehabilitation (18%) resulted in significant improvements in QoL over the year.

**Conclusions:**

Melanoma diagnosis leads to a marked QoL reduction, particularly in lower stage women, with most patients improving over time. However, substantial intra‐individual variation emphasizes the need for regular QoL assessments. Further research is needed to assess the long‐term effectiveness of psycho‐oncological support and rehabilitation.

**Trial Registration:**

German Clinical Trials Register No. DRKS00010005, 08. March 2016

## 1 Introduction

The number of cases of cutaneous melanoma is increasing in most countries, resulting in significant morbidity and mortality. By 2040, the global incidence of melanoma is projected to increase to 510,000 new cases and 96,000 deaths, a more than 50% rise [1]. Between 1970 and 2020, the age‐standardized incidence rate of melanoma in Germany increased from 3 to 19 cases per 100,000 population per year. In 2020, melanoma ranked forth (women) and fifth (men) among the most common solid tumors in Germany (excluding non‐melanoma skin cancer), with a median age of 63 years for women and 69 years for men. It is one of the most common cancers in the 20–40 age group [2].

The diagnosis of melanoma can significantly impact patients' health‐related quality of life (HRQoL) due to physical, emotional, and social challenges associated with the disease and its treatment. QoL can influence patient prognosis, including survival [3, 4, 5, 6, 7, 8]. Many melanoma patients experience ongoing physical symptoms that affect their QoL. Treatments such as surgery, or systemic therapy such as immunotherapy can lead to side effects like pain, lymphedema and fatigue, which particularly impact physical functioning and overall health perception [9, 10]. Furthermore, a melanoma diagnosis often triggers emotional distress, anxiety, and depression, with patients worrying about cancer recurrence and the stress of ongoing medical treatments [10, 11]. However, there is also evidence that even brief interventions may be sufficient to reduce stress and improve HRQoL [12].

To address these challenges, the current guideline on melanoma of the Association of the Scientific Medical Societies in Germany [13] recommends the initiation of psycho‐oncological care and rehabilitation measures in primary care. In Germany, all melanoma patients are eligible for rehabilitation services, which are covered by health insurance. However, melanoma patients often constitute a minority in oncology rehabilitation facilities, which can lead to feelings of displacement and discomfort when confronted with other seriously ill patients with different types of cancer [14, 15]. In 2022, only a small fraction of newly diagnosed melanoma patients in Germany (1554 out of approximately 25,000) received preventive care or rehabilitation in larger specialized facilities [16].

Among cancer patients in general, up to 40% may report moderate‐to‐high levels of unmet supportive care needs, and around 25%–84% frequently express dissatisfaction with the lack of appropriate information. These unmet needs can significantly impact patient QoL [17, 18]. A cross‐sectional multisite survey of 472 UK melanoma patients (stages I to III) found that 27% experienced unmet support needs in the mid‐to‐long term (three months up to five years) following primary treatment, especially among younger, less educated, distressed, or socially isolated individuals [17]. A large study in Denmark revealed that 21.4% of cancer patients reported an unmet need for counseling, and 18.8% reported an unmet need for physical rehabilitation shortly after diagnosis (2–5 months). Interestingly, the frequency of these unmet needs was lower in melanoma patients, at 17.8% for counseling and 9.1% for rehabilitation [19].

To our knowledge, there is limited research on how QoL is affected shortly after diagnosis and how it evolves thereafter. Most previous studies measured QoL at a single point in time with different surveys. In particular, there is a paucity of data pertaining to the QoL of patients with early‐stage melanoma (AJCC Stages 0–II). This is likely attributable to the favorable prognosis and less invasive treatments associated with the early stages of melanoma in comparison to other cancers [20]. A survey of melanoma survivors conducted in Australia revealed that a significant number of individuals reported persistent QoL issues, concerns about cancer recurrence, and unmet information needs up to five years after diagnosis [21]. In addition, the extent to which patients use services such as psycho‐oncology counseling or rehabilitation has not been well studied.

Therefore, our study aimed to describe how QoL changes in melanoma patients within the first year after diagnosis. Specifically, our hypothesis was that tumor stage, sex, and aftercare services (psycho‐oncological measures, inpatient and outpatient rehabilitation) may influence QoL. We used different questionnaires to assess QoL.

## 2 Methods

### 2.1 Study Design, Setting and Participants

This single‐center, prospective cohort study was conducted at the Department of Dermatology, Fachklinik Hornheide. Patients aged 18–65 with stage 0 to IIIC melanoma (based on 2009 AJCC guidelines [22]) diagnosed within the past six weeks were eligible. This timeframe was chosen to capture patients' experiences soon after diagnosis while allowing sufficient time for scheduling initial clinic visits. The baseline visit was scheduled at the time of enrollment, which could occur any time within six weeks of diagnosis. Exclusion criteria included insufficient German language skills or inability to complete questionnaires independently. Of 229 invited, 221 patients were enrolled (Supporting Information S1: Figure 23S). The study was registered in the German Clinical Study Registry (DRKS00010005) [23] and adheres to STROBE guidelines [24].

### 2.2 Survey Instruments and Data Management

Between 2016 and 2018, patients completed electronic questionnaires at baseline and at 3, 6, 9, and 12 months, either at clinic visits or from home. Data were pseudonymized and securely transferred to a GDPR‐compliant registry via the MoPat system [25], with MoPat@Home [26] enabling web‐based follow‐up. Physicians documented clinical data in electronic case report forms within MoPat, with prior training and a documentation test.

Routine clinical data (age, sex, diagnosis, therapy), and patient‐reported outcomes (general well‐being [WHO‐5], HRQoL [FACT‐M], and psycho‐oncologic care needs [Hornheider Screening Instrument ‐ HSI]) were collected. Comorbid conditions were assessed using a structured questionnaire adapted from the Work Ability Index [27]. Patients were asked to indicate the presence of specific medical conditions, which were categorized by organ system.

The WHO‐5 [28] is a 5‐item tool for general well‐being. A percentage value below 52% suggests possible depression or high stress. The FACT‐M (Version 4) [29, 30] is a 51‐item tool measuring HRQoL and covers physical, social/family, emotional, and functional well‐being domains, with additional questions concerning melanoma‐specific well‐being. Scores range from 0 to 172, where higher scores indicate better HRQoL. The HSI [31] is a 7‐item tool screening for psychological stress and care needs, with scores ranging from 0 to 14. A score of four or more indicates a need for psycho‐oncologic care. For ease of comparison across instruments in figures, all scores were transformed to a percentage scale, where the maximum possible score corresponds to 100% (e.g., FACT‐M 172 = 100%). For the FACT‐M, changes of 5% were considered clinically meaningful, based on previously published estimates for subscales of the FACT‐M [32].

At each follow‐up, patients also reported the use of psychological support or inpatient and outpatient rehabilitation services. Details of these services were not part of the record. For full questionnaire see Supplement.

### 2.3 Statistics

Data were analyzed using the statistical program R (version 4.1.4., ggplot2 library). A sample size of 220 patients was estimated (see Supplement). Descriptive statistics were calculated for all clinical parameters and survey questionnaires. Outcome measures were general well‐being and HRQoL. Statistical tests for associations between variables were based on a two‐sided significance level of 5%. Regarding missing values, no imputation was performed (Supporting Information S1: Table 10S). Non‐parametric tests were used (no assumption of normally distributed data), specifically Wilcoxon tests (unpaired or paired) or Kruskal‐Wallis tests. Spearman correlation coefficient was calculated to assess association of metric variables. Pearson's Chi‐squared Test was applied to analyze tabular data. There was no adjustment for multiple testing (non‐confirmatory observational study). Efforts were made to minimize potential sources of bias (see Supplement).

## 3 Results

### 3.1 Characteristics of Patient Cohort

Median age of all 221 patients was 51 (19–65). Women had a younger median age than men (*p* = 0.011) (Supporting Information S1: Table 1S and [23]). Of all patients, 193 (87%) completed all five visits (Supporting Information S1: Figure 23S).

Median tumor thickness (Breslow, AJCC 2009) was 0.8 mm (range 0.1–15 mm; Interquartile range (IQR) 0.4–1.4 mm). Eight patients (4%) were initially diagnosed with two melanomas (detailed melanoma characteristics: Supporting Information S1: Table 2S).

A total of 500 surgeries were performed on 221 patients, with a median of 2 procedures per patient (range 1–6, IQR 2–2). Diagnostic and therapeutic procedure details are outlined in Supporting Information S1: Table 3S.

### 3.2 The Impact of Melanoma on General Well‐Being, HRQoL, and Need for Psycho‐Oncological Care

At baseline, 123 (56%) patients were stage 0/IA, 68 (31%) stage IB/IIA, and 30 (14%) ≥ stage IIB (Supporting Information S1: Table 4S, [23]). After one year, 172 (90%) patients remained at the same stage, and 20 (9%) progressed. There was no sex difference in stage (*p* = 0.66) (Supporting Information S1: Table 5S).

**FIGURE 1:**
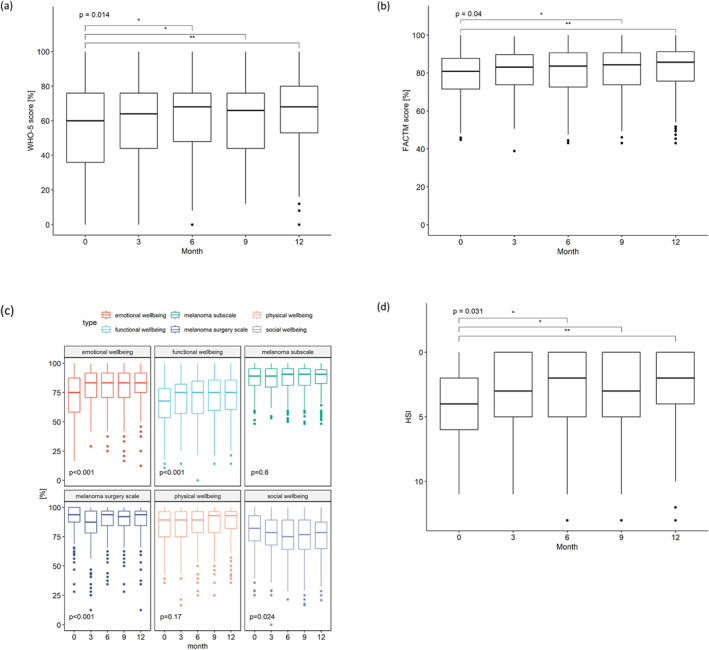
(a) General well‐being in all patients, measured with the WHO‐5. A score below 52% indicates the presence of a stressful condition in need of treatment. (b) HRQoL in all patients, measured with the FACT‐M. Higher scores represent better HRQoL. (c) HRQoL subscales in all patients, measured with the FACT‐M. *p*‐values are provided for each subscale. (d) Need for psycho‐oncological care in all patients, measured with the HSI. A score of 4 and above indicates that the patient needs psycho‐oncological care.

Median WHO‐5 well‐being rose from 60% (range 0%–100%; IQR 36%–76%) to 68% (range 0%–100%; IQR 52%–80%) at 12 months (*p* = 0.002; Figure 1a), with a significant improvement from the 6th month onwards (*p* = 0.014). At baseline, 84 (38%) patients were below the threshold of 52%. HRQoL measured by FACT‐M improved from a median of 139 (range 77–172; IQR 123–151) to 147 (range 74–172; IQR 127–156; *p* = 0.01; Figure 1b). Emotional and functional subscales dipped initially, the melanoma surgery subscale at 3 months, and social well‐being at 6 months, while physical domains remained stable (Figure 1c). After one year, 111 (58%) patients reported numbness at the surgery site, and 49 (26%) residual swelling (FACT‐M ‐ Melanoma surgical Subscale).

Shortly after diagnosis, the median HSI score was 4 (range 0–11, IQR 2–6), indicating a psycho‐oncological care need for half of the patients. This need decreased significantly within a year (median 2, range 0–13, IQR 0–5) (*p* = 0.002) (Figure 1d). HSI item 2 (“How have you felt emotionally in the last three days?”) showed the highest improvement after 12 months (average −0.5 points).

Comorbidities did not affect general well‐being or HRQoL, except for patients with mental illness (e.g., depression) (*n* = 45), who had lower HRQoL at baseline (median 114 vs. 144, *p* < 0.001). More than two surgeries reduced the melanoma‐surgery‐subscale score from month three after diagnosis (median 25.5 vs. 29, *p* = 0.001; for other periods see supplement). Adjuvant interferon therapy was associated with lower HRQoL from month three (median 129 vs. 147, *p* < 0.001). General anesthesia led to a slight HRQoL decrease at month nine (median 143 vs. 149, *p* = 0.028). Age, education, family history, and melanoma location had no impact.

FACT‐M and WHO‐5 were strongly correlated (*R* = 0.76, *p* < 0.001), with WHO‐5 explaining 58% of FACT‐M's variance. FACT‐M and HSI showed similar correlation (*R* = 0.73, *p* < 0.001, 53% variance explained; Supporting Information S1: Figure 21S and 22S).

### 3.3 The Impact of Tumor Stage on General Well‐Being, HRQoL, and Need for Psycho‐Oncological Care

HRQoL varied widely even among patients with small tumors (FACT‐M: Supporting Information S1: Figure 24Sa; emotional well‐being subscale: Supporting Information S1: Figure 24Sb). This variation persisted across all visits and was consistent in WHO‐5 and HSI scores (data not shown).

**FIGURE 2:**
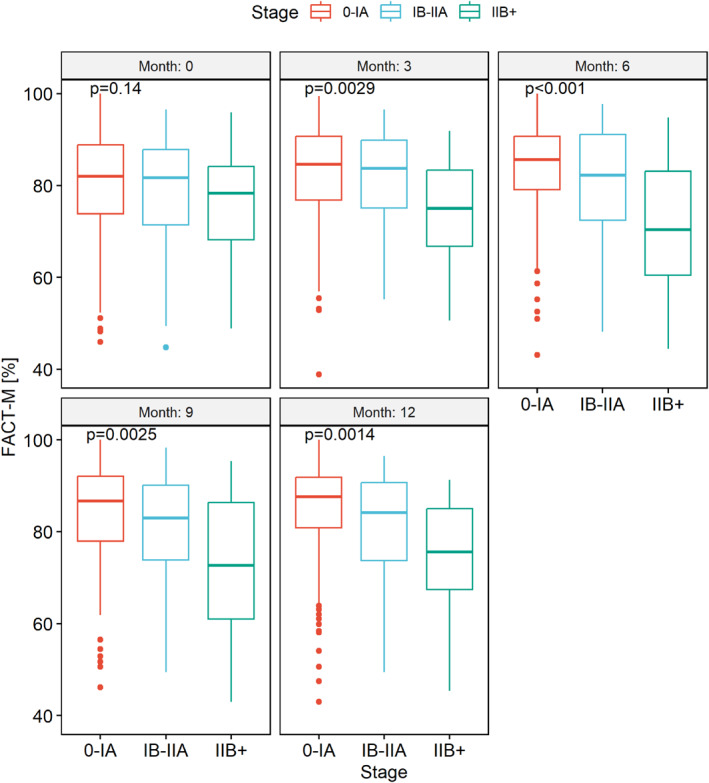
FACT‐M, shown for each time period, in relation to tumor stage. No differences regarding age (*p* = 0.906) or sex (*p* = 0.908) were detected between tumor stages.

Starting after 3 months, general well‐being and HRQoL significantly declined in patients with higher tumor stages, beginning at stage IB/IIA (Figure 2; for WHO‐5: Supporting Information S1: Figure 1S).

Unlike general well‐being and HRQoL, the need for psychological support (HSI) showed no significant differences across tumor stages (Supporting Information S1: Figure 2S).

## 4 Sex Differences in General Well‐Being, HRQoL, and Need for Psycho‐Oncological Care

**FIGURE 3:**
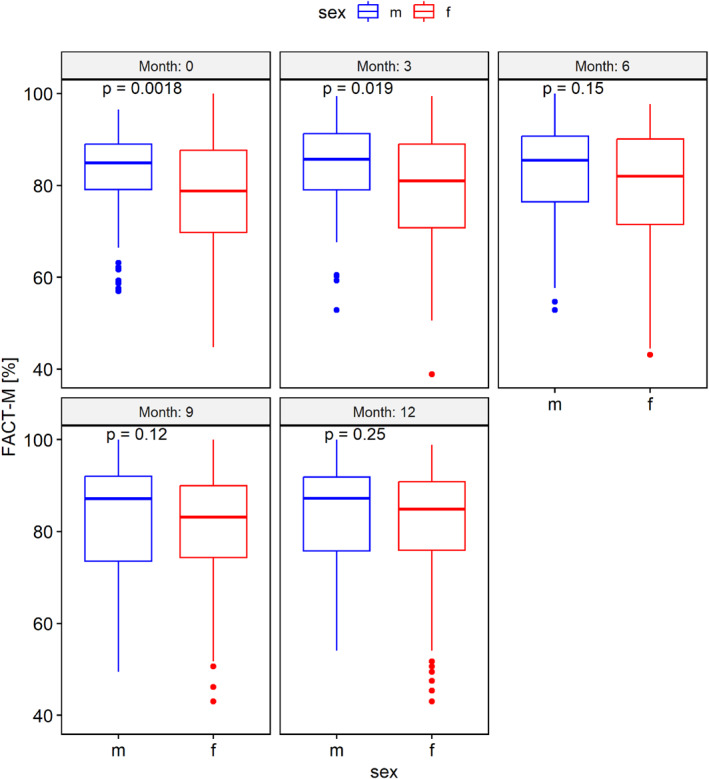
FACT‐M for men and women, shown for each period.

At baseline and three months, women (*n* = 138) had significantly lower general well‐being (52% vs. 64%, *p* = 0.010) and HRQoL (135.5 vs. 146, *p* = 0.002) than men (*n* = 83); these differences resolved by 12 months (WHO‐5 68% vs. 72%, *p* = 0.38; FACT‐M 145 vs. 148.2, *p* = 0.26) (Figure 3 and Supporting Information S1: 3S).

Throughout the year, women had significantly worse QoL than men on the FACT‐M emotional, general‐melanoma, and physical well‐being subscales, with the largest difference in physical well‐being at early visits. The melanoma‐surgery subscale differed only at 12 months. No sex difference was found in functional well‐being (Supporting Information S1: Figure 5S–10S).

**FIGURE 4:**
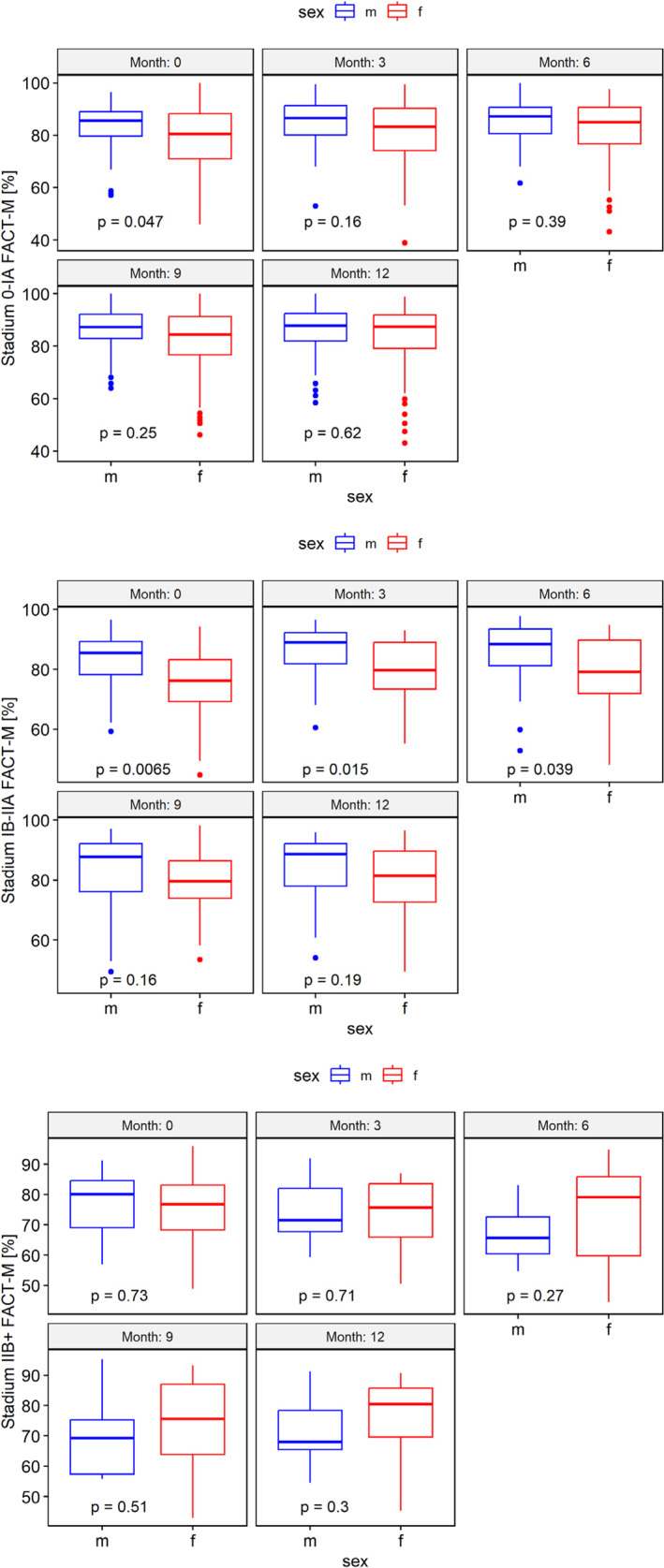
FACT‐M for men and women, differentiated by tumor stage, shown for each period.

Women also had a higher need for psychological support, particularly in the first quarter (*p* < 0.001) (Supporting Information S1: Figure 4S).

In terms of tumor stage, women with stage 0/IA or IB/IIA experiences worse general well‐being and HRQoL at baseline; IB/IIA differences persisted up to six months (Figure 4 and Supporting Information S1: 11S). Regarding the subscales, women with stage 0/IA or IB/IIA had significantly lower physical well‐being and general‐melanoma scale scores. In Addition, women with stage IB/IIA had lower emotional, functional well‐being, and melanoma surgery scale scores (Supporting Information S1: Figure 13S–18S).

Women with stage 0/IA or IB/IIA had a significantly higher need for psychological support throughout the year (Supporting Information S1: Figure 12S).

No sex differences were found for stage ≥ IIB melanoma.

### 4.1 Trajectories of QoL

**FIGURE 5:**
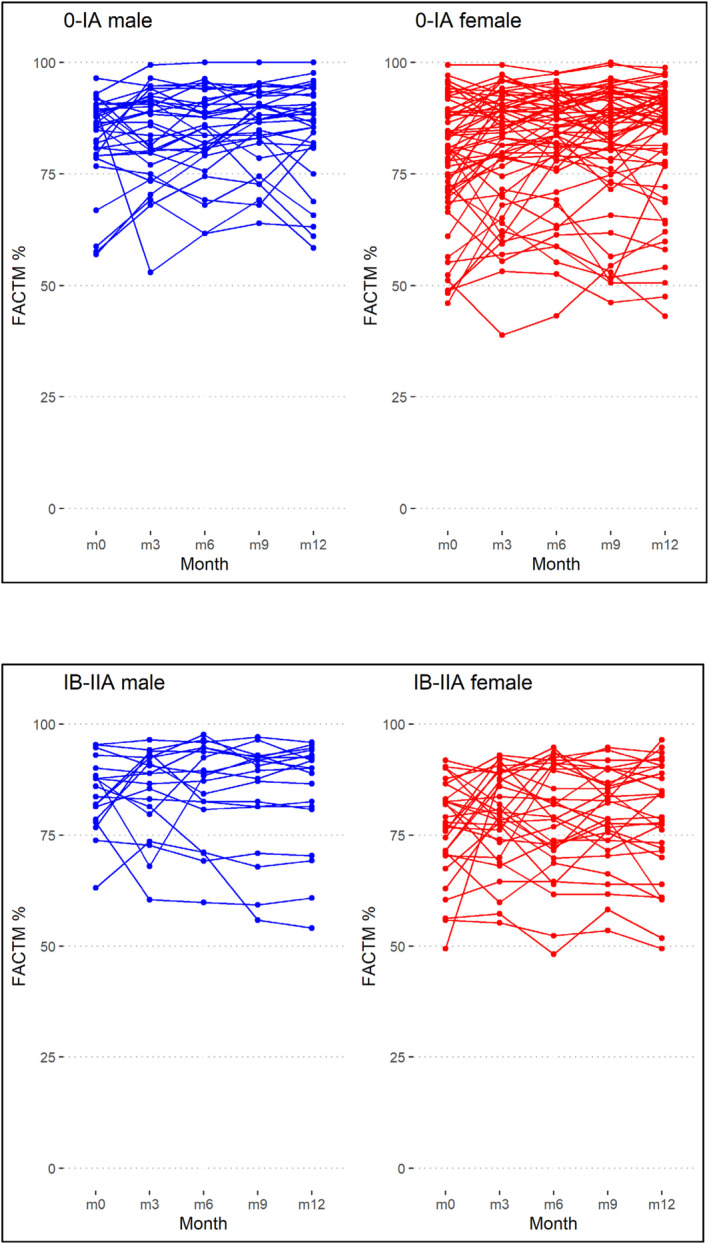
Trajectories of FACT‐M over the year, for women and men with different tumor stages.

HRQoL varied widely over 12 months, with some patients experiencing little change and others showing both increases and decreases. The median HRQoL variation within a single patient was 11.1% (range 0%–40.7%, IQR 6.2%–16.3%). The greatest variance between patients was seen at baseline, particularly in patients with stage 0/IA melanoma, where 54% had their worst HRQoL at this time. The generally poorer HRQoL of women with stage IB/IIA melanoma is also highlighted here (Figure 5).

### 4.2 The Impact of Psycho‐Oncological Consultation and Rehabilitation on General Well‐Being, HRQoL, and Need for Psycho‐Oncological Care

Fifty‐three patients (24%) attended a psycho‐oncological consultation, primarily those with thicker melanomas, higher tumor stages and more comorbidities (Supporting Information S1: Table 6S). These patients had significantly worse QoL and HRQoL at baseline and study end, with no improvement over 12 months (Supporting Information S1: Table 8S and Figure 19S). Lower‐stage patients received less psycho‐oncological counseling (*p* < 0.001). Of the 45 patients with a pre‐existing mental health condition, only 19 (42%) received consultation.

Forty patients (18%) underwent rehabilitation (35 inpatients, 6 outpatients, including one who received both). Similarly, the patients had thicker melanomas, higher tumor stages and more comorbidities (Supporting Information S1: Table 7S). At baseline, rehabilitation patients had worse QoL, HRQoL, and higher psychological care needs, with no improvement over one year (Supporting Information S1: Table 9S and Figure 20S).

Patients with thicker tumors and more comorbidities were more likely to receive psycho‐oncological consultation or rehabilitation (*p* < 0.001).

## 5 Discussion

Our study highlights several critical insights into QoL among melanoma patients after diagnosis, particularly concerning stage‐related and sex‐specific differences, psychosocial support needs, and clinical implications.

Many patients suffered from reduced QoL at baseline (within six weeks after diagnosis), with 38% scoring below the WHO‐5 threshold of 52%, indicative of poor well‐being; however, QoL improved for most within a year. Women had significantly lower HRQoL than men, even in early stages, indicating greater need for psychosocial support, possibly influenced by sociocultural factors. Previous studies have similarly identified sex‐based differences [33, 34, 35, 36, 37], linking them to distinct coping strategies and societal expectations. Although the improvement in WHO‐5 scores over 12 months was statistically significant, the median change of eight points remained below the frequently cited 10‐point threshold for clinical relevance [28]. However, this threshold is based on convention rather than empirical validation and was originally applied in psychiatric settings [28]. To date, no minimal clinically important difference has been established for cancer or melanoma populations, and interpretation should therefore remain cautious.

### 5.1 Clinical Implications

An important finding was the variability in QoL trajectories. While some recovered, others experienced ongoing distress or worsening QoL. This intra‐individual variability supports the necessity of repeated QoL assessments, as earlier studies have also noted dynamic changes in patient experiences [36, 38]. Stage ≥ IB patients showed lower HRQoL and higher psycho‐oncological care needs, likely due to invasive procedures like sentinel lymph node biopsy and frequent monitoring [11, 30]. Our study excluded patients with stage IV disease, whose treatment and prognosis differ significantly. However, even patients with small tumor thicknesses (< 1 mm) experienced considerable variability, challenging assumptions that favorable prognoses correlate with minimal psychological impact, as other studies have also observed distress in localized melanoma cases [35, 38].

Most QoL research predominantly focuses on advanced melanoma or specific treatments [11, 30, 39, 40, 41, 42]; studies on early‐stage cases and longitudinal outcomes are limited. Consistent with our findings, Finnish research reported no significant QoL differences in long‐term localized melanoma survivors compared to the general population [43], aligning with other studies [37, 44]. In contrast, a Romanian study found persistent QoL declines, but small sample sizes and lack of stage‐specific data limit comparisons [41]. Regarding tumor thickness, international findings vary. While some link greater thickness to reduced QoL [45, 46], others found negligible differences [33, 34, 47, 48, 49]. These inconsistencies highlight the need for further research.

Our results indicate no HRQoL differences by melanoma location except mucosal cases, whose HRQoL notably declined. Though limited by sample size, this aligns with findings that anatomical locations affect outcomes due to surgical or functional considerations [50, 51]. Many of our patients experienced long‐term sensory disturbances (58%) or swelling (26%) post‐surgery, demonstrating that physical sequelae can extend well beyond the acute treatment phase and echoing studies on long‐term physical sequelae [46].

Fear of recurrence, sociodemographic factors, and specific treatments influence distress. A German study identified younger age, employment, and interferon therapy as predictors of distress, independent of tumor stage or localization [52]. Similarly, UK research found higher anxiety among female patients and those quickly referred to clinics, emphasizing psychological complexity [36]. Our findings support these observations and highlight the importance of addressing psychosocial needs in both early and advanced‐stage patients. In contrast to our findings, Mehnert et al. reported depressive symptoms in 24% of cancer patients, with melanoma patients showing the lowest rates among all tumor entities and no sex‐specific differences [51]. However, their study included various tumor types, including stage IV disease, where higher psychological burden is expected. Moreover, melanoma patients represented only 2% of their cohort, limiting conclusions about this specific group. Focusing exclusively on stages 0–III melanoma, our study offers a more stage‐specific and longitudinal view, revealing lower overall distress but clear sex‐specific differences in HRQoL and psychosocial needs.

Despite clear indications for psycho‐oncological care in half the patients, uptake was unexpectedly low, with only 24% receiving counseling. Even among patients with pre‐existing mental health issues, only 42% received consultation. Research suggests that melanoma patients often face barriers to accessing appropriate psychological interventions, reflecting a potential gap in addressing psychosocial needs [52, 53]. The observed improvement of HSI score likely reflects psychological adaptation to the initial diagnosis, completion of surgical treatment, and increased familiarity with follow‐up routines. Furthermore, ongoing medical care and improved information over time may have contributed to reduced uncertainty and perceived stress.

Only 18% of patients in our cohort participated in rehabilitation, despite full insurance coverage. This low uptake may reflect that many early‐stage melanoma patients do not perceive themselves as “ill enough,” alongside practical barriers such as work commitments, limited program availability, and complex application procedures. Psychological factors, including stigma or preference for informal support, may also contribute. While psycho‐oncological consultation and rehabilitation measures appeared to have limited effectiveness in improving HRQoL, our small sample size (53 resp. 40 patients) likely reduced the statistical power to detect meaningful effects. Though one small study showed that structured interventions reduce distress [12], others found no effect [54]. This emphasizes the need for research into tailored interventions, including whether pharmacological approaches like antidepressants or anxiolytics might complement counseling for some patients [53].

Short instruments like the WHO‐5 or HSI correlated well with the longer HRQoL scale (FACT‐M), although they are not interchangeable. However, their brevity makes them practical tools for integrating QoL screening into routine clinical care, supporting earlier detection of distress. Digital integration of such tools could enhance early detection and ongoing monitoring, addressing calls in the literature for improved QoL assessment methods [55, 56, 57]. This approach would enable clinicians to allocate support more efficiently, especially to patients with fluctuating QoL metrics who might otherwise go unnoticed.

Our study has several strengths: It provides a unique longitudinal perspective on HRQoL in melanoma patients, especially early‐stage patients often underrepresented in research. By incorporating both short and comprehensive QoL instruments, the study not only validates the utility of tools like WHO‐5 and HSI but also offers practical solutions for routine clinical implementation. The exploration of sex‐specific differences adds to the growing evidence for gender‐sensitive approaches in psycho‐oncological care, while the identification of underutilized services highlights a critical area for improvement. Finally, the study's findings regarding intra‐individual QoL variability emphasize the need for personalized, adaptive interventions.

### 5.2 Study Limitations

This study has certain limitations: As a single‐center cohort study, our findings may not be fully generalizable. Older patients (> 65 years) were excluded due to parallel assessments of work ability [23], limiting applicability to this demographic. Furthermore, the study lacked detailed records on the type and duration of psycho‐oncological interventions, precluding a more granular analysis of their efficacy. Selection bias is another concern, as patients with higher tumor stages were more likely to discontinue participation, reducing the power of subgroup analyses. Additionally, the study used the AJCC 2009 version to classify melanomas. In order to maintain comparability over the period, the 2017 version was not changed during the ongoing study. Also, effective adjuvant treatment options have been available for stage IIB/C and III since 2017 [58, 59]. These changes likely impact QoL outcomes in ways not captured by our data.

### 5.3 Conclusion

This study underscores the importance of targeted psychosocial support for melanoma patients, particularly women and those in early stages experiencing high distress. The underutilization of psycho‐oncological services highlights an urgent need to improve accessibility and tailor interventions to meet diverse patient needs. The integration of brief QoL assessments, such as the WHO‐5 or HSI, into routine practice offers a promising approach for identifying at‐risk patients and delivering timely support. Future research should prioritize stage‐specific interventions, explore alternative approaches for high‐distress patients, and evaluate digital tools for QoL monitoring. By addressing these gaps, clinicians can better support melanoma patients in managing distress and improving long‐term outcomes.

## Funding

The study was funded by a grant from Deutsche Rentenversicherung (Grant 622‐4012). The funder had no role in the design, data collection, data analysis, and reporting of this study.

## Ethics Statement

This study was performed in line with the principles of the Declaration of Helsinki. Approval was granted by the local Ethics Committee (Ethics Committee Westfalen‐Lippe, number: 2016–069‐f‐S).

## Consent

Written informed consent was obtained from all participants before enrollment in the study. The authors affirm that human research participants provided informed consent for publication.

## Conflicts of Interest

The authors declare no conflicts of interest.

## Supporting information


Supporting Information S1
