# Supplementary material for: Gender Differences in Quality of Life and Psycho‐Oncological Needs During the First Year After Melanoma Diagnosis
Source: Psychooncology. 2025 Nov 19;34(11):e70335. doi: 10.1002/pon.70335 (PMC12629850; doi:10.1002/pon.70335)
Supplement: Supplementary file 1 — Supporting Information S1 [file PON-34-e70335-s001.docx]

**Online Supplementary Material**

**Gender Differences in Quality of Life and Psycho-Oncological Needs**

**During the First Year After Melanoma Diagnosis**

Susanne Dugas-Breit, Jessica Hassel, Martin Dugas^*^, Hans-Joachim Schulze^*^

* These authors share last authorship.

Corresponding Author:
Susanne Dugas-Breit
Department of Dermatology, Heidelberg University Hospital, Heidelberg, Germany
susanne.dugas-breit@med.uni-heidelberg.de

**Table 1S** Patient population characteristics at baseline

|  | **Number** | | **Median Age (range)** | |  |
| --- | --- | --- | --- | --- | --- |
| All Patients | 221 | | 51 (19-65) | |  |
| Female | 138 (62%) | | 50 (21-65) | |  |
| Male | 83 (38%) | | 54 (19-65) | |  |
| **Relationship Status** | **Partnership** | **Single** | **Separated** | **Widowed** |  |
|  | 166 (76%) | 33 (15%) | 19 (9%) | 3 (2%) |  |
| **School Education** | **9 Years** | **10 Years** | **Baccalaureate** | **University Degree** | **Other** |
|  | 39 (18%) | 85 (38%) | 51 (23%) | 41 (19%) | 5 (2%) |
| **Family history of cancer** | **No** | **Yes** | **Thereof melanoma** | |  |
|  | 51 (23%) | 170 (77%) | 42 (25%) | |  |
| **Health insurance** | **Public** | **Private** | | |  |
|  | 186 (84%) | 35 (16%) | | |  |

**Table 2S** Melanoma characteristics

|  |  | **Number** |
| --- | --- | --- |
| **Total** |  | 229^§^ |
| **Type** | Superficial spreading | 143 (62%) |
|  | Nodular | 25 (11%) |
|  | Lentigo maligna-melanoma | 12 (5%) |
|  | Acrolentiginous | 10 (4%) |
|  | Amelanotic | 2 (1%) |
|  | Unspecified or other | 37 (16%) |
| **Location** | Lower extremities and hip | 77 (34%) |
|  | Torso | 71 (31%) |
|  | Upper extremities and shoulder | 36 (16%) |
|  | Face, head and neck | 25 (11%) |
|  | Unknown | 2 (1%) |
|  | Mucosal | 2 (1%) |
|  | Missing value | 16 (7%) |

^§^ 221 patients had 229 melanomas

**Table 3S** Diagnostic and therapeutic measures performed

|  |  | **Number** |
| --- | --- | --- |
| **Device Diagnostics** | Lymph node sonography | 210 (95%) |
|  | Abdominal sonography | 110 (50%) |
|  | Chest X-ray | 108 (49%) |
|  | Whole-body CT scan | 40 (18%) |
|  | Cranial MRI | 32 (15%) |
|  | PET-CT | 2 (1%) |
|  | Scintigraphy | 2 (1%) |
| **Surgery Detail** | Total procedures | 500 (100%) |
|  | Local anesthesia | 367 (73%) |
|  | General anesthesia | 133 (27%) |
|  | Re-excisions for larger safety margin | 223 (45%) |
|  | Surgery for metastases | 26 (12%) |
|  | Sentinel lymph node biopsy | 97 (44%) |
|  | Subsequent lymphadenectomy | 25 (11%) |
| **Conservative Therapy** | Adjuvant therapy with interferon-alpha | 40 (18%) |
|  | Enrolled in a therapy trial | 2 (1%) |
|  | Adjuvant radiation | 3 (1%) |

**Table 4S** Tumor stage of patients at first and last visit

| **Tumor Stage** | **First Visit** | **Last Visit** |
| --- | --- | --- |
| 0 | 12 (5.5%) | 11 (5.7%) |
| IA | 111 (50.2%) | 92 (48.1%) |
| IB | 51 (22.8%) | 43 (22.4%) |
| IIA | 17 (7.8%) | 12 (6.7%) |
| IIB | 10 (4.6%) | 4 (1.9%) |
| IIC | 9 (4.1%) | 4 (1.9%) |
| IIIA | 3 (1.4%) | 10 (5.2%) |
| IIIB | 7 (3.2%) | 10 (5.2%) |
| IIIC | 1 (0.5%) | 3 (1.4%) |
| IV | 0 (0%) | 3 (1.4%) |

**Table 5S** Tumor stage of men and women at first visit

| **Tumor Stage** | **male** | **female** |
| --- | --- | --- |
| 0 | 6 | 6 |
| IA | 39 | 72 |
| IB | 20 | 31 |
| IIA | 7 | 10 |
| IIB | 3 | 7 |
| IIC | 3 | 6 |
| IIIA | 0 | 3 |
| IIIB | 4 | 3 |
| IIIC | 1 | 0 |

FACT-M melanoma surgery subscale scores and *P*-values from visit 2: Comparison of patients who had more than 2 surgical procedures with patients who had fewer procedures:
visit 2: median 25.5 versus 29; *P*=0.001
visit 3: median 27 versus 30; *P*=0.003
visit 4: median 28 versus 30; *P*<0.001
visit 5: median 27 versus 30; *P*=0.016

**Table 6S** Characteristics of patients with (n=53) / without (n=168) psycho-oncological consultation

|  | **Psycho-oncological consulation** | | | | | |  |
| --- | --- | --- | --- | --- | --- | --- | --- |
|  | **With** | | | **Without** | | | ***P*-Value** |
| **Sex** | Male 16 (30%) / Female 37 (70%) | | | Male 67 (40%) / Female 101 (60%) | | | 0.2550 |
|  | **Median** | **Range** | **IQR** | **Median** | **Range** | **IQR** |  |
| **Age** | 50 | 28-65 | 44-57 | 52 | 19-65 | 45-58 | 0.5715 |
| **Number of comorbidities** | 2.5 | 0-9 | 1-4 | 2 | 0-10 | 1-3 | 0.0498 |
| **Melanoma tumor thickness**  **(mm)** | 1.5 | 0.3-15 | 0.7-2.9 | 0.6 | 0.1-15 | 0.4-1.1 | <0.001 |
| **Tumor stage** | **0/IA** | **IB/IIA** | **≥IIB** | **0/IA** | **IB/IIA** | **≥IIB** |  |
|  | 17 (32%) | 20 (38%) | 16 (30%) | 106 (63%) | 48 (29%) | 14 (8%) | <0.001 |

**Table 7S** Characteristics of patients with (n=40) / without (n=181) rehabilitation

|  | **Rehabilitation** | | | | | |  |
| --- | --- | --- | --- | --- | --- | --- | --- |
|  | **With** | | | **Without** | | | ***P*-Value** |
| **Sex** | Male 17 (43%) / Female 23 (57%) | | | Male 66 (37%) / Female 115 (68%) | | | 0.5891 |
|  | **Median** | **Range** | **IQR** | **Median** | **Range** | **IQR** |  |
| **Age** | 54.5 | 33-64 | 49-59 | 50 | 19-65 | 42-58 | 0.079 |
| **Number of comorbidities** | 3 | 0-10 | 2-4 | 1 | 0-10 | 1-3 | <0.001 |
| **Melanoma tumor thickness**  **(mm)** | 1.34 | 0.3-15 | 0.9-2.2 | 0.6 | 0.1-15 | 0.4-1.2 | <0.001 |
| **Tumor stage** | **0/IA** | **IB/IIA** | **≥IIB** | **0/IA** | **IB/IIA** | **≥IIB** |  |
|  | 9 (22%) | 23 (58%) | 8 (20%) | 114 (63%) | 45 (25%) | 22 (12%) | <0.001 |

**Table 8S** WHO-5 (%), FACT-M and HSI Scores for patients with (n=53) / without (n=168) psycho-oncological consultation

|  | **Psycho-oncological consulation** | | | | | | | | |
| --- | --- | --- | --- | --- | --- | --- | --- | --- | --- |
|  | **With** | | | | **Without** | | | |  |
| **WHO-5** | Median | Range | IQR | Mean | Median | Range | IQR | Mean | *P*-value |
| Visit 1 | 48 | 0-88 | 24-62 | 45.49 | 64 | 0-100 | 36-80 | 57.94 | 0.001 |
| Visit 5 | 58 | 12-88 | 43-72 | 54.91 | 72 | 0-100 | 60-80 | 65.85 | 0.001 |
| *P*-value | 0.009 | | | | 0.012 | | | | |
| **FACT-M** |  |  |  |  |  |  |  |  |  |
| Visit 1 | 124 | 77-157 | 110-137 | 123.1 | 143 | 83-172 | 132.8-153 | 140.1 | <0.001 |
| Visit 5 | 134 | 85-164 | 110-148 | 130 | 151 | 74-172 | 136-158 | 143.2 | <0.001 |
| *P*-value | 0.076 | | | | 0.015 | | | | |
| **HSI** |  |  |  |  |  |  |  |  |  |
| Visit 1 | 6 | 0-11 | 4-8 | 5.89 | 3 | 0-11 | 1-5 | 3.23 | <0.001 |
| Visit 5 | 4 | 0-13 | 2-6 | 4.13 | 2 | 0-12 | 0-4 | 2.79 | 0.004 |
| *P*-value | 0.008 | | | | 0.070 | | | | |

**Table 9S** WHO-5 (%), FACT-M and HSI Scores for patients with (n=40) / without (n=181) rehabilitation

|  | **Rehabilitation** | | | | | | | | |
| --- | --- | --- | --- | --- | --- | --- | --- | --- | --- |
|  | **With** | | | | **Without** | | | |  |
| **WHO-5** | Median | Range | IQR | Mean | Median | Range | IQR | Mean | *P*-value |
| Visit 1 | 52 | 0-96 | 34-64 | 49.23 | 64 | 0-100 | 36-80 | 56.27 | 0.080 |
| Visit 5 | 54 | 0-92 | 38-73 | 53.11 | 72 | 8-100 | 60-80 | 66.03 | 0.003 |
| *P*-value | 0.22 | | | | <0.001 | | | | |
| **FACT-M** |  |  |  |  |  |  |  |  |  |
| Visit 1 | 133 | 84-157 | 108-142 | 127.3 | 141 | 77-172 | 126-153 | 138.0 | 0.003 |
| Visit 5 | 133 | 78-160 | 110-152 | 129.5 | 149 | 74-172 | 134-158 | 143.6 | 0.004 |
| *P*-value | 0.50 | | | | <0.001 | | | | |
| **HSI** |  |  |  |  |  |  |  |  |  |
| Visit 1 | 5 | 0-11 | 2.75-8 | 5.08 | 3 | 0-11 | 1-6 | 3.61 | 0.005 |
| Visit 5 | 4 | 0-12 | 2-6 | 4.30 | 2 | 0-13 | 0-4 | 2.82 | 0.005 |
| *P*-value | 0.44 | | | | 0.003 | | | | |

**Table 10S** Completeness of FACT-M, WHO-5 and HSI Scores by Visit.
Numbers of patients for each score at each visit are provided.

|  | **FACT-M** | **WHO-5** | **HSI** |
| --- | --- | --- | --- |
| Visit 1 | 221 | 216 | 219 |
| Visit 2 | 188 | 190 | 188 |
| Visit 3 | 187 | 189 | 185 |
| Visit 4 | 186 | 182 | 187 |
| Visit 5 | 190 | 190 | 191 |

**Table 11S** WHO-5 score by month. Results from Figure 1a in table format.

|  | **Min.** | **Q1** | **Median** | **Q3** | **Max.** |
| --- | --- | --- | --- | --- | --- |
| Month 0 | 0% | 36% | 60% | 76% | 100% |
| Month 3 | 0% | 44% | 64% | 76% | 100% |
| Month 6 | 0% | 48% | 68% | 76% | 100% |
| Month 9 | 12% | 44% | 66% | 76% | 100% |
| Month 12 | 0% | 53% | 68% | 80% | 100% |

**Table 12S** FACT-M score by month. Results from Figure 1b in table format.

|  | **Min.** | **Q1** | **Median** | **Q3** | **Max.** |
| --- | --- | --- | --- | --- | --- |
| Month 0 | 44.7% | 71.5% | 80.9% | 87.8% | 100% |
| Month 3 | 38.9% | 73.8% | 83.1% | 89.7% | 99.4% |
| Month 6 | 43.1% | 72.6% | 83.7% | 90.6% | 100% |
| Month 9 | 43.0% | 73.8% | 84.4% | 90.7% | 100% |
| Month 12 | 43.0% | 75.7% | 85.7% | 91.2% | 100% |

**Table 13S** HIS score by month. Results from Figure 1d in table format.

|  | **Min.** | **Q1** | **Median** | **Q3** | **Max.** |
| --- | --- | --- | --- | --- | --- |
| Month 0 | 0 | 2 | 4 | 6 | 11 |
| Month 3 | 0 | 0 | 3 | 5 | 11 |
| Month 6 | 0 | 0 | 2 | 5 | 13 |
| Month 9 | 0 | 0 | 3 | 5 | 13 |
| Month 12 | 0 | 0 | 2 | 4 | 13 |

**Fig. 1S** WHO-5, shown for each visit, in relation to tumor stage


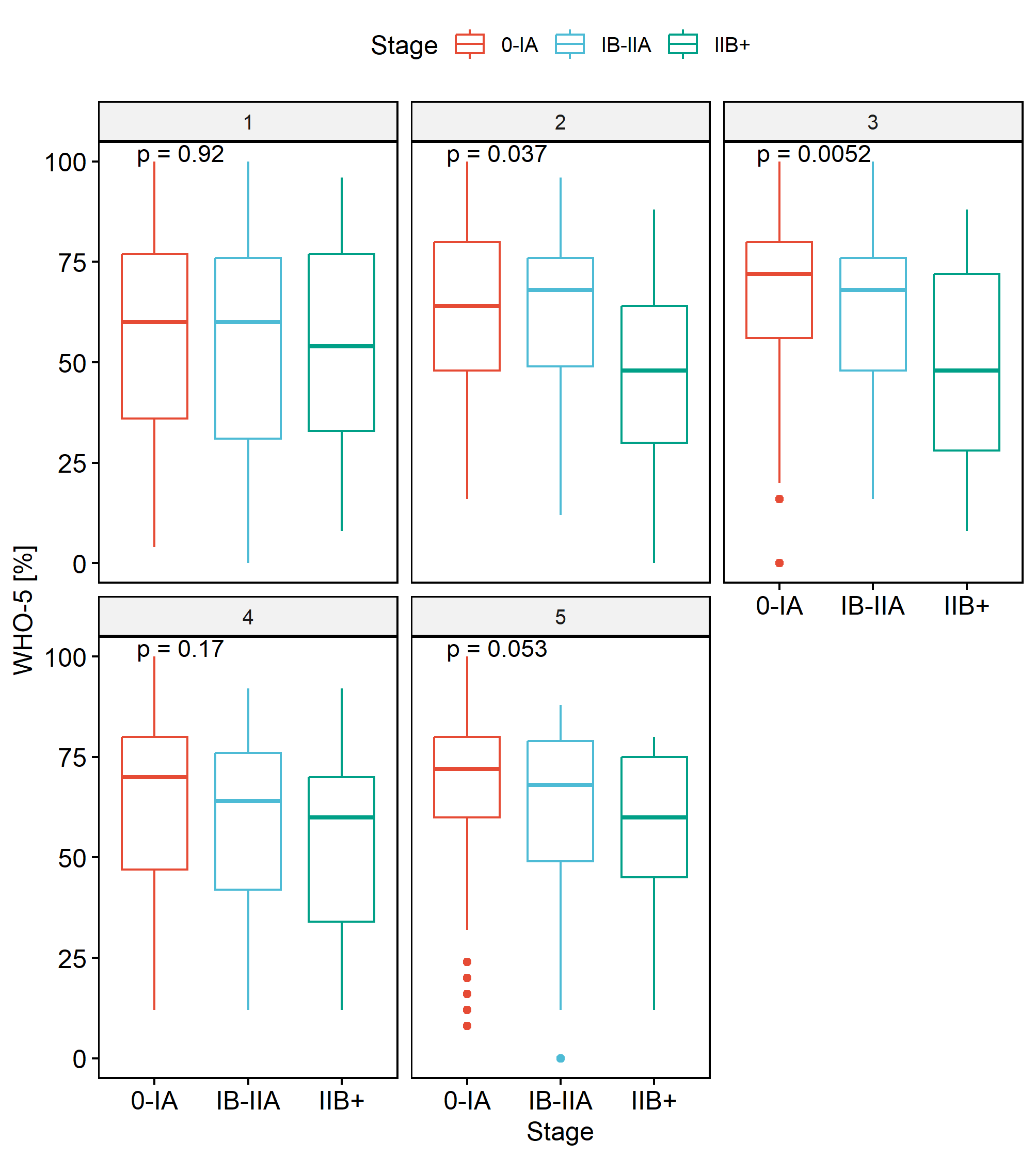


**Fig. 2S** HSI, shown for each visit, in relation to tumor stage


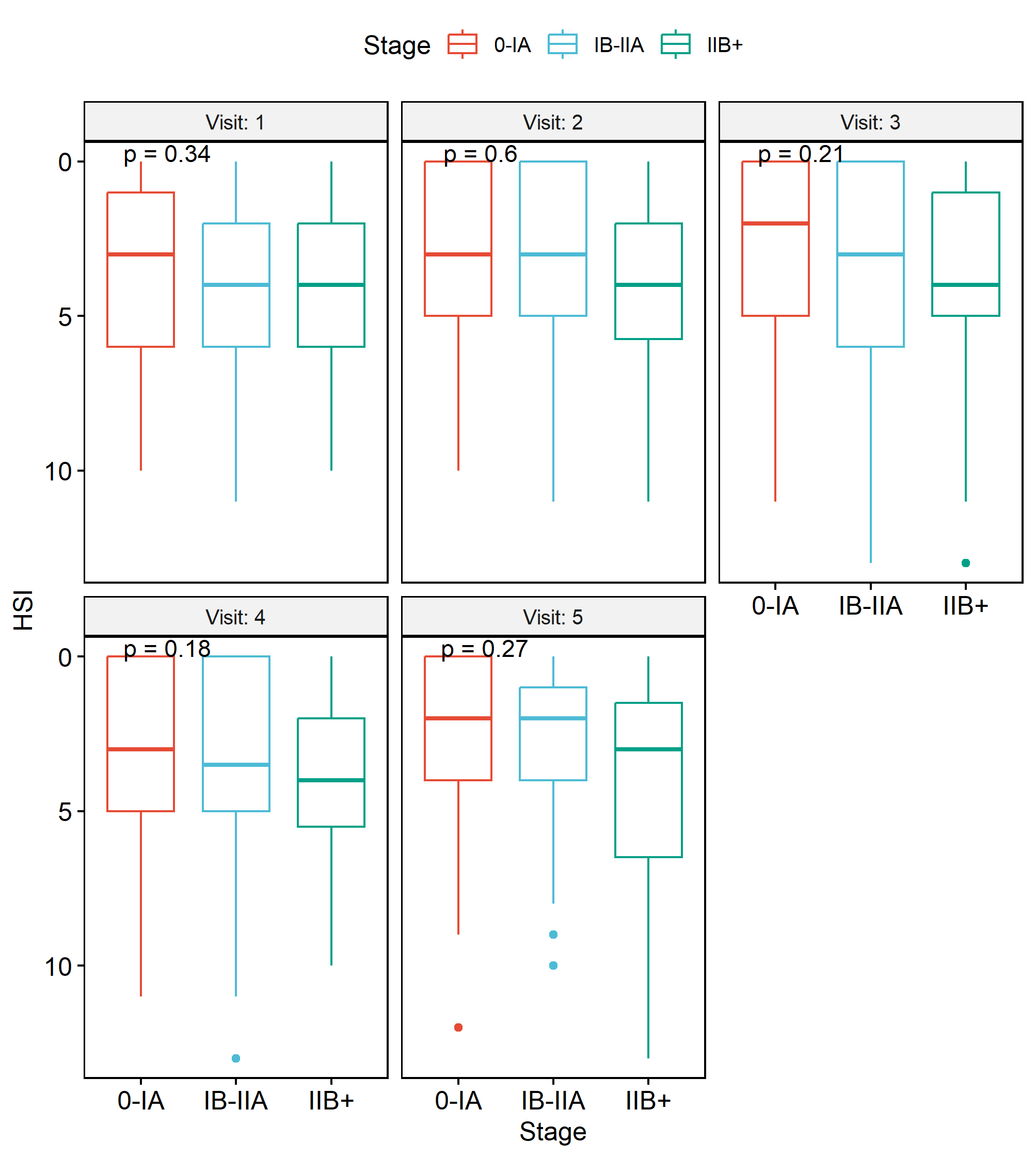


**Fig. 3S** WHO-5 in men (n = 83) and women (n = 138)


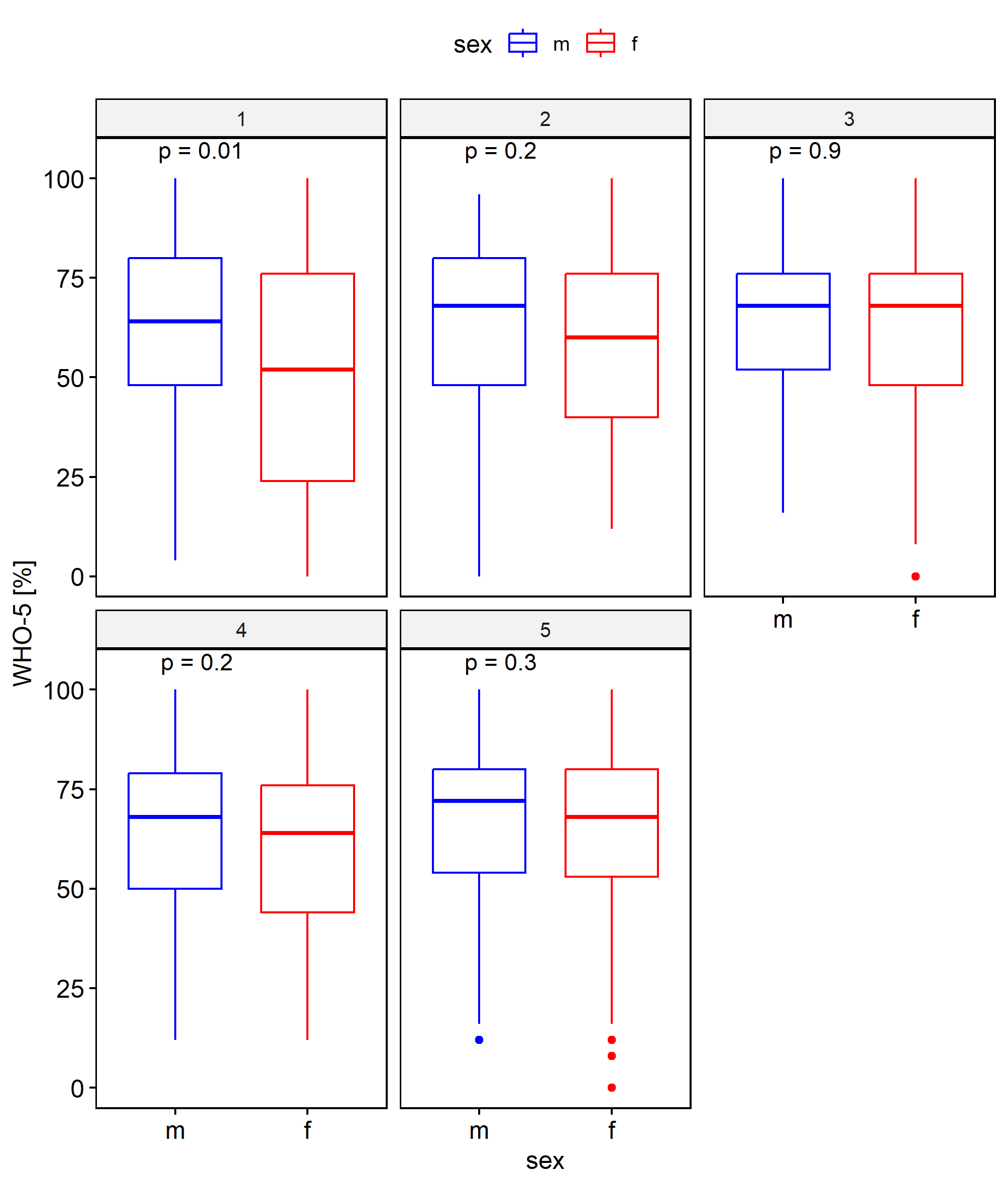


**Fig. 4S** HSI in men (n = 83) and women (n = 138)


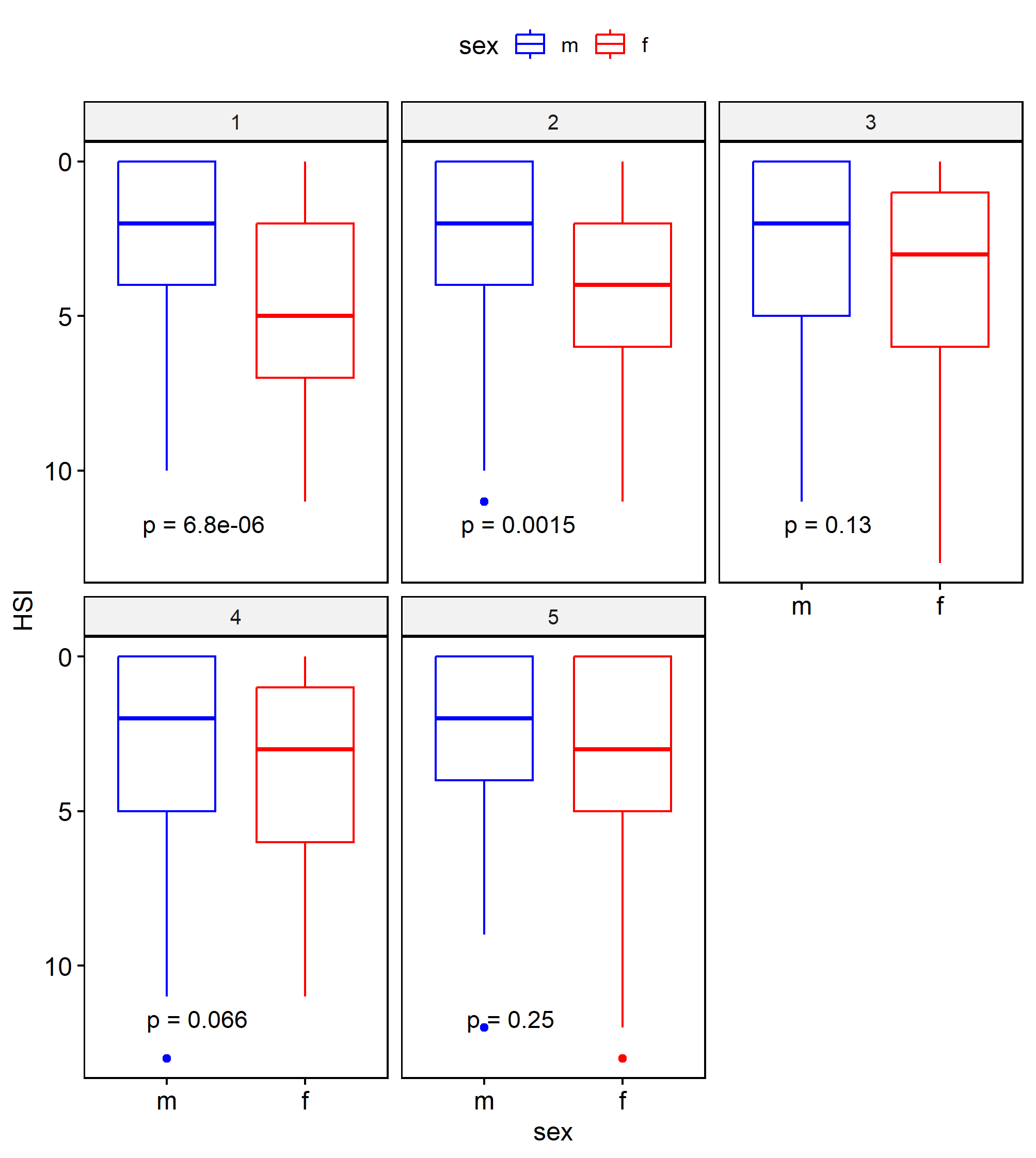


**Fig. 5S** FACT-M Subscale Emotional Well-being for men and women


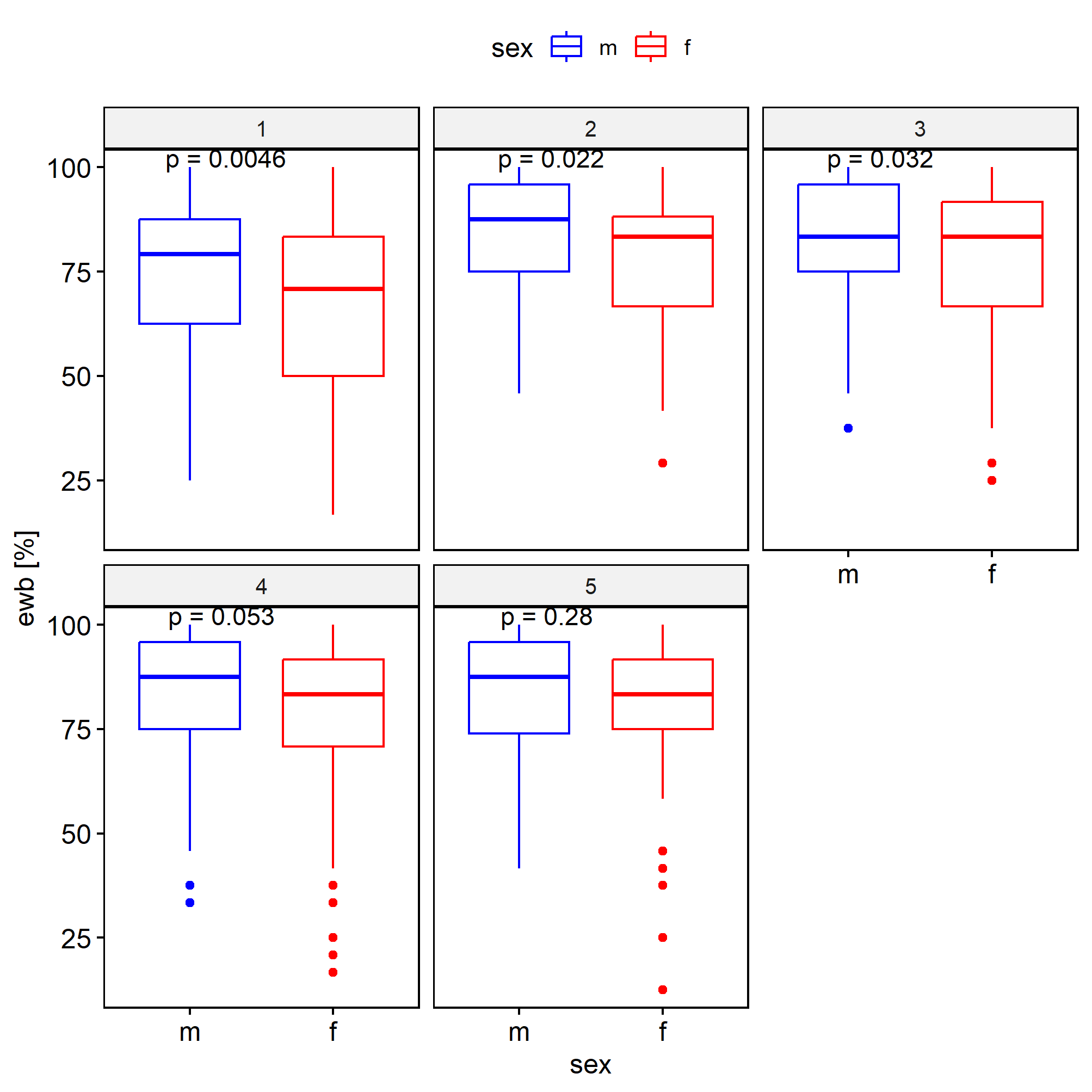


**Fig. 6S** FACT-M Subscale Functional Well-being for men and women


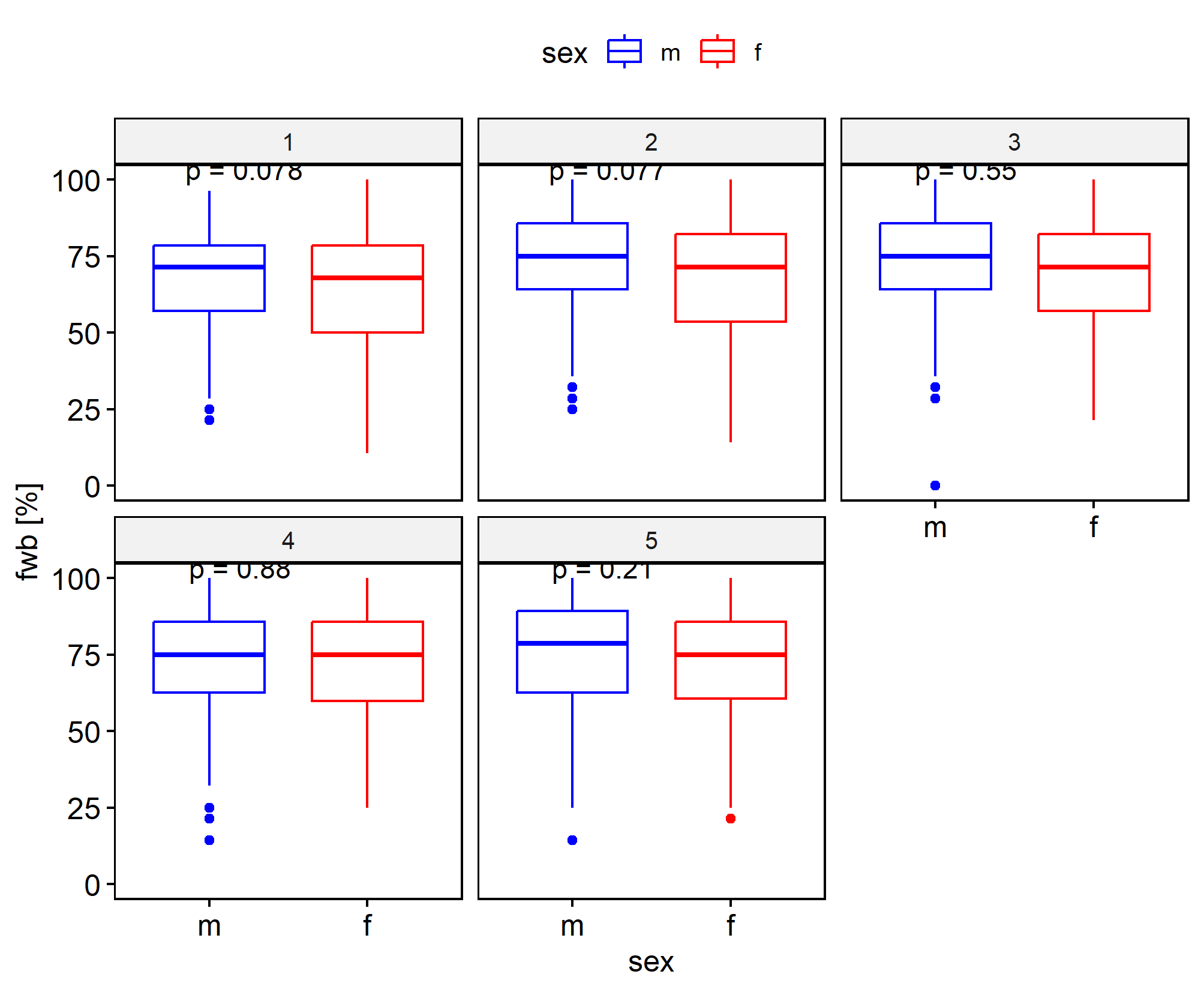


**Fig. 7S** FACT-M Melanoma Subscale for men and women


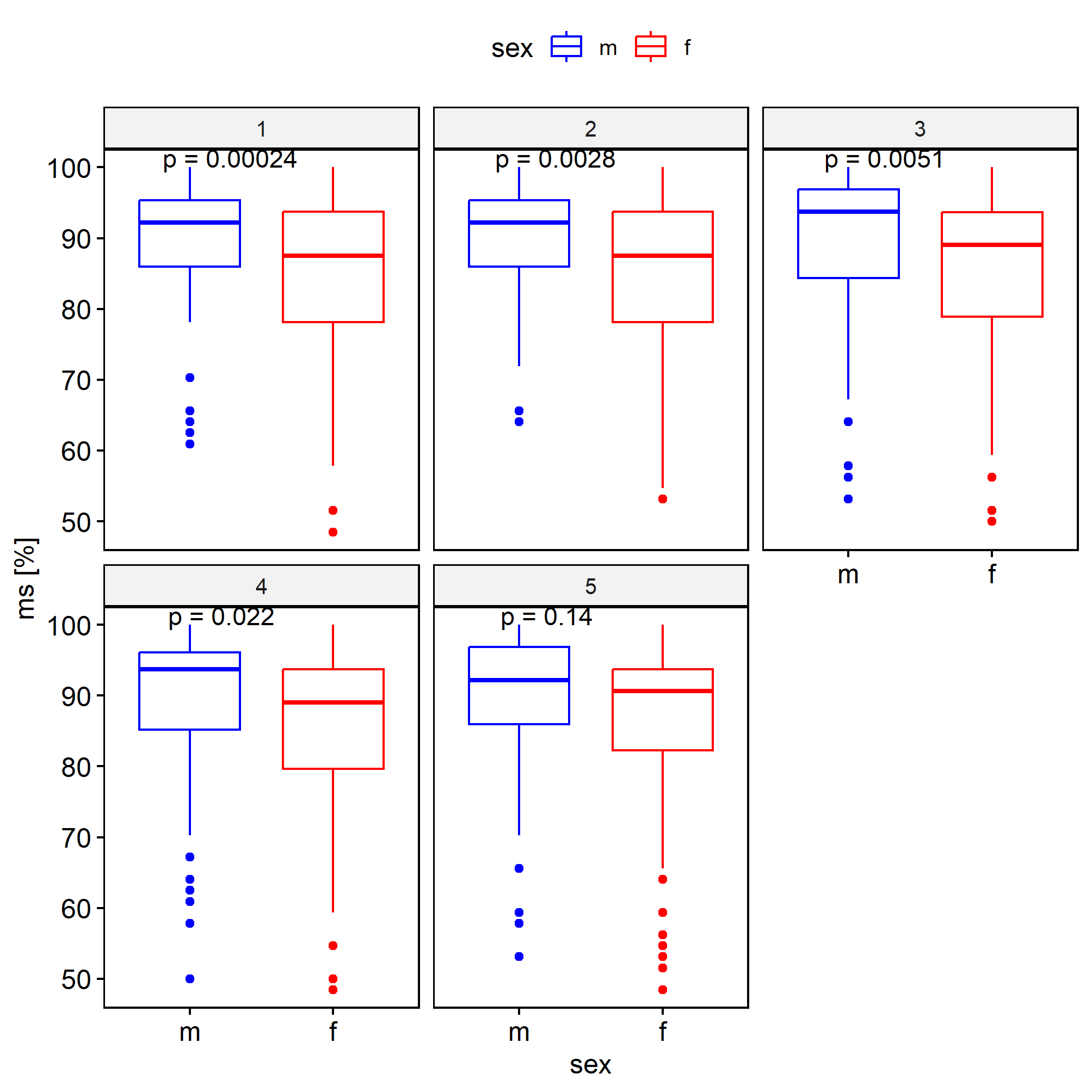


**Fig. 8S** FACT-M Melanoma Surgery Subscale for men and women


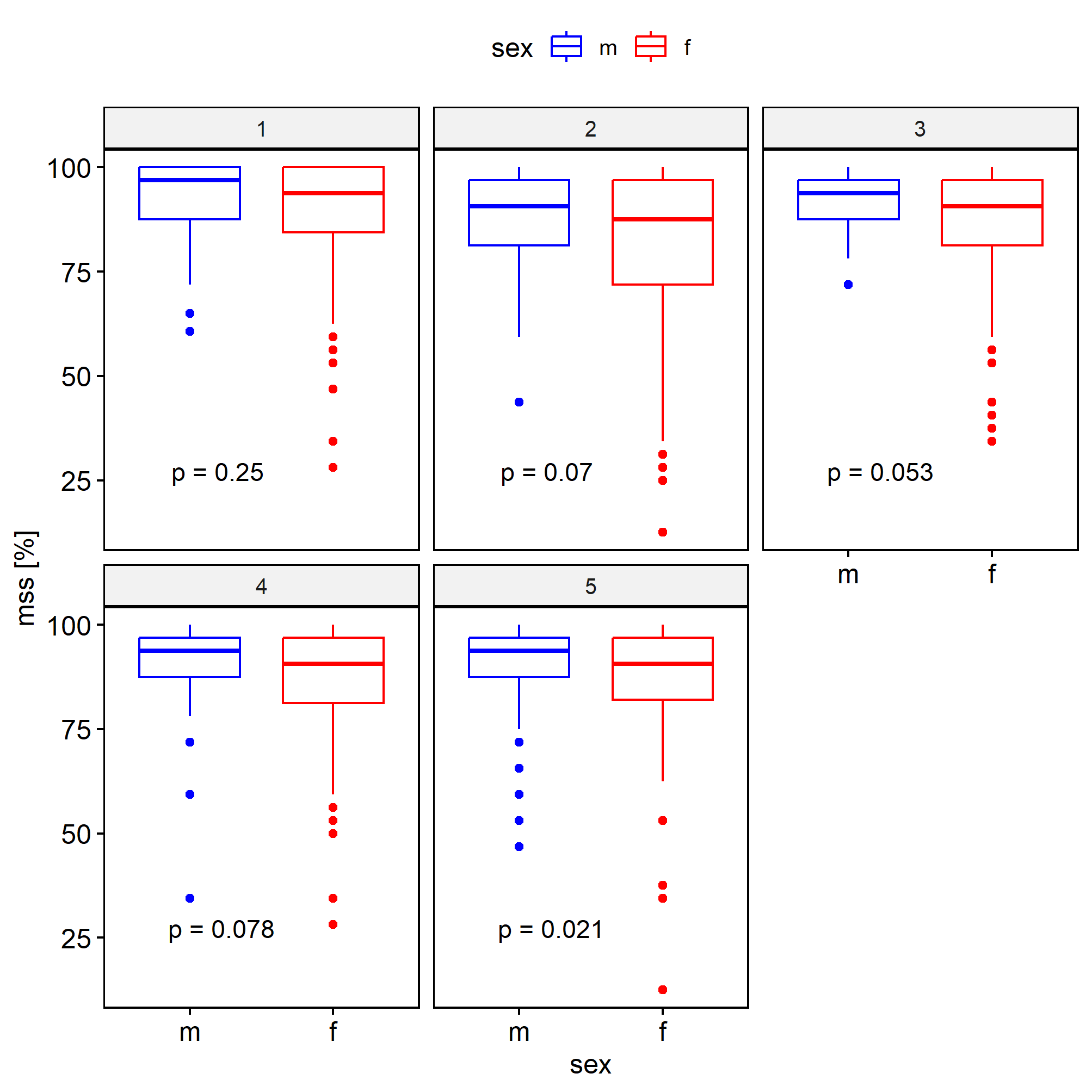


**Fig. 9S** FACT-M Subscale Physical Wellbeing for men and women


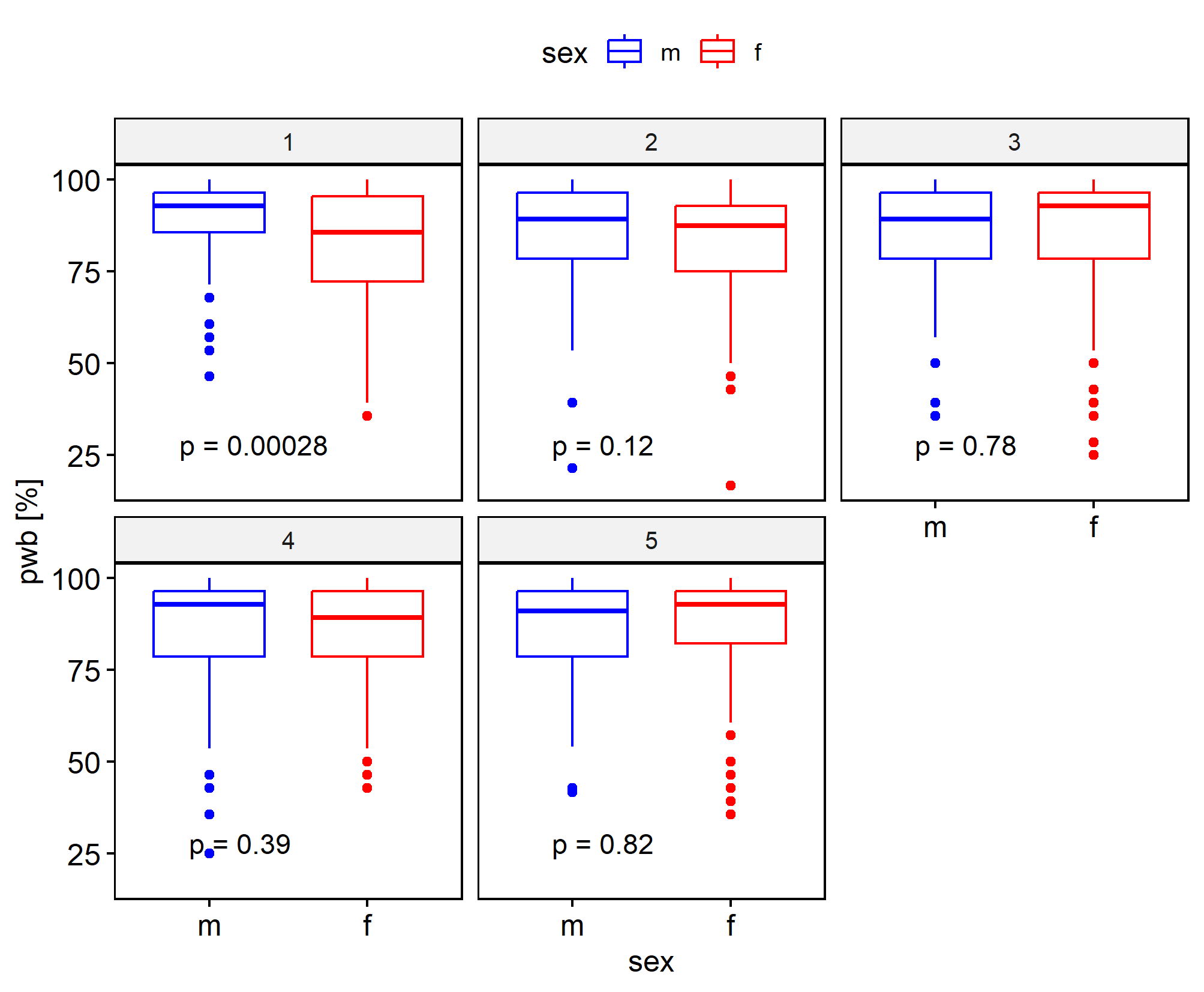


**Fig. 10S** FACT-M Subscale Social Wellbeing for men and women


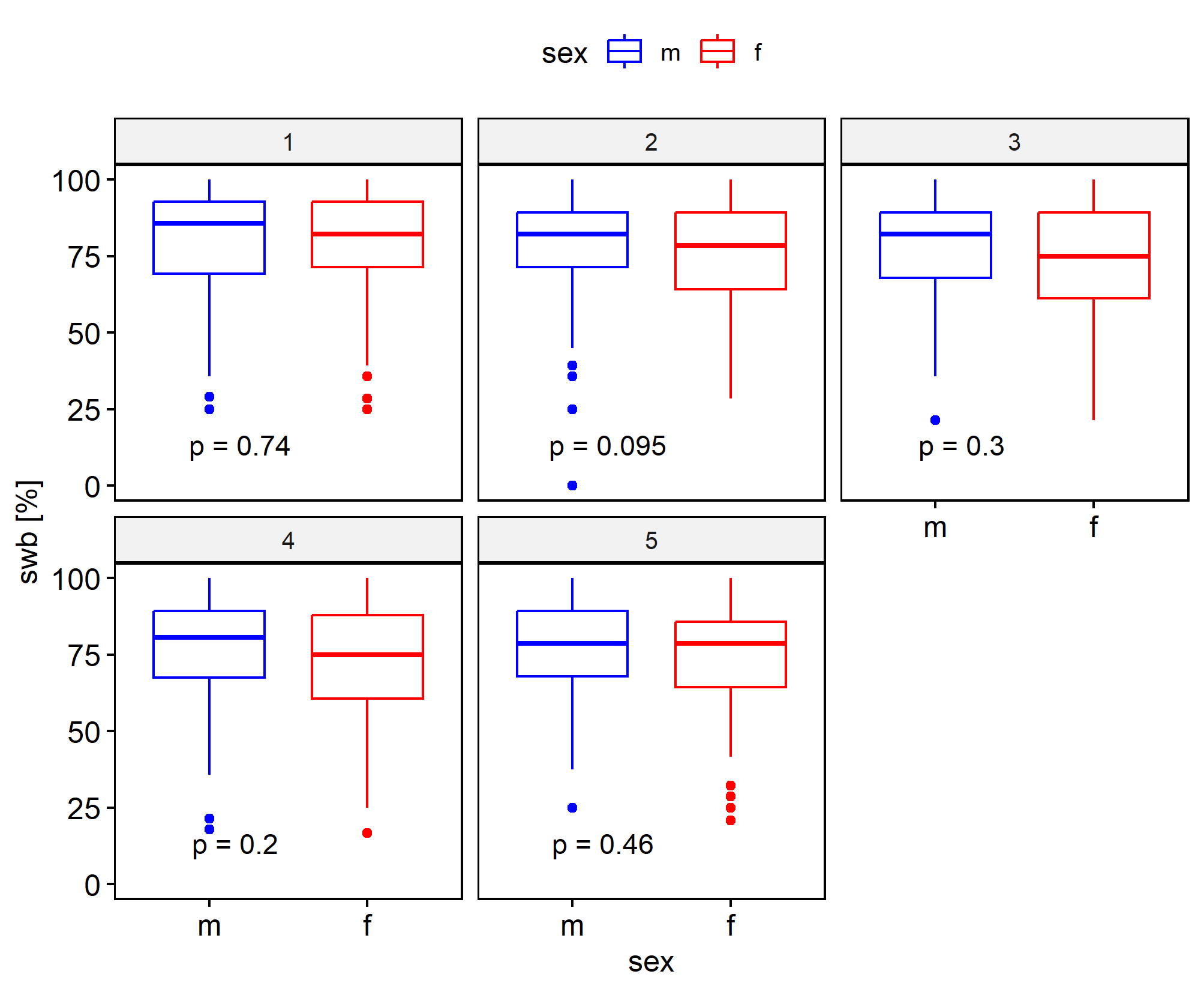


**Fig. 11S** WHO-5 for men and women, differentiated by tumor stage, shown for each visit


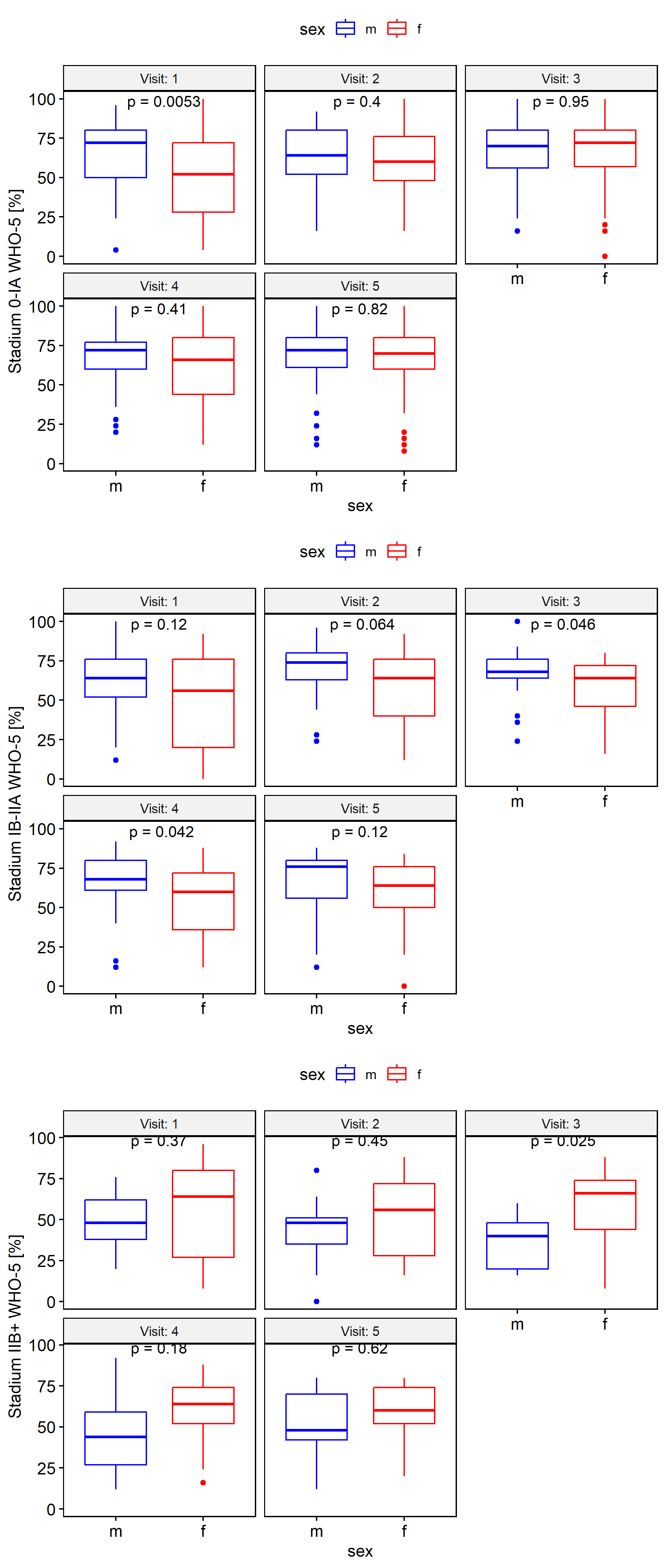


**Fig. 12S** HSI for men and women, differentiated by tumor stage, shown for each visit


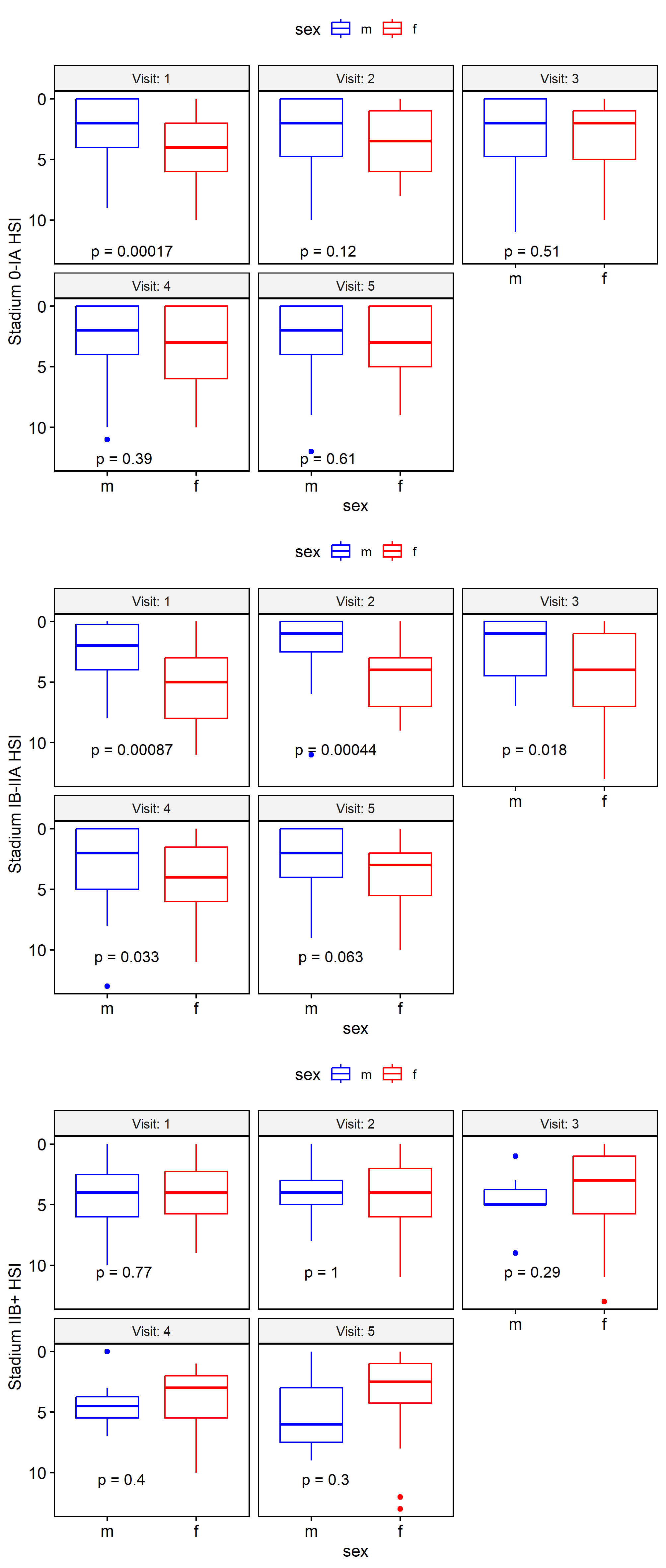


**Fig. 13S** FACT-M Subscale Emotional Well-being for men and women, differentiated by tumor stage, shown for each visit


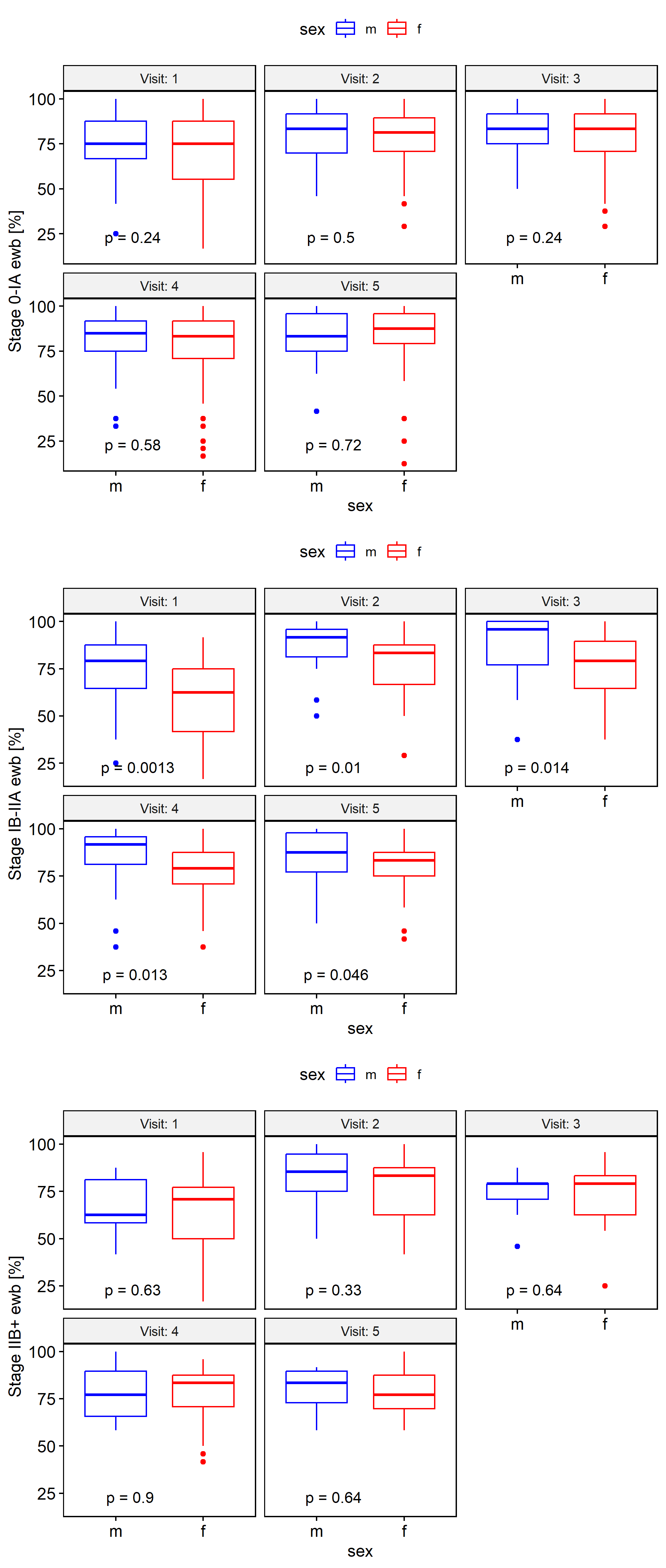


**Fig. 14S** FACT-M Subscale Physical Well-being for men and women, differentiated by tumor stage, shown for each visit
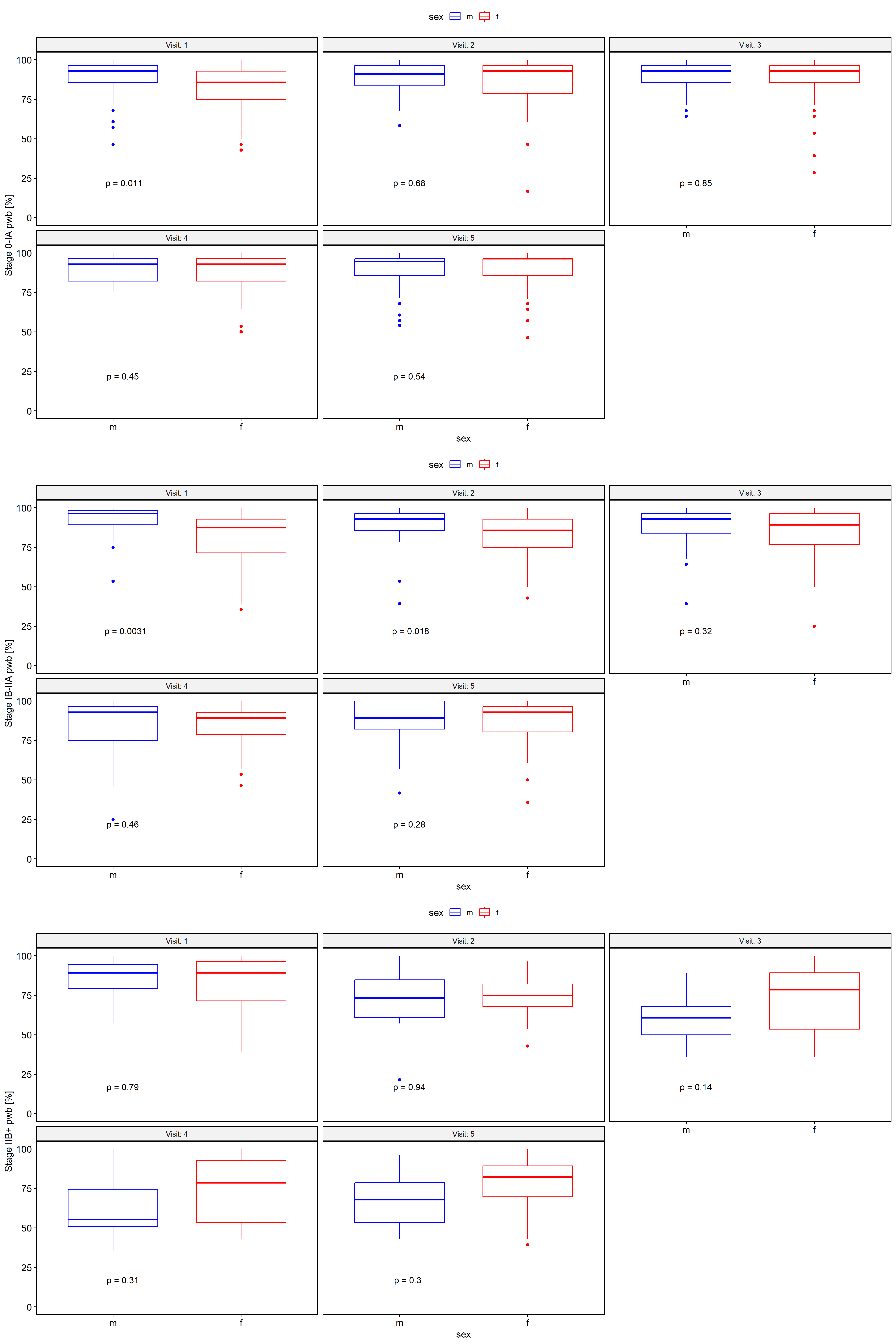


**Fig. 15S** FACT-M Subscale Social Well-being for men and women, differentiated by tumor stage, shown for each visit


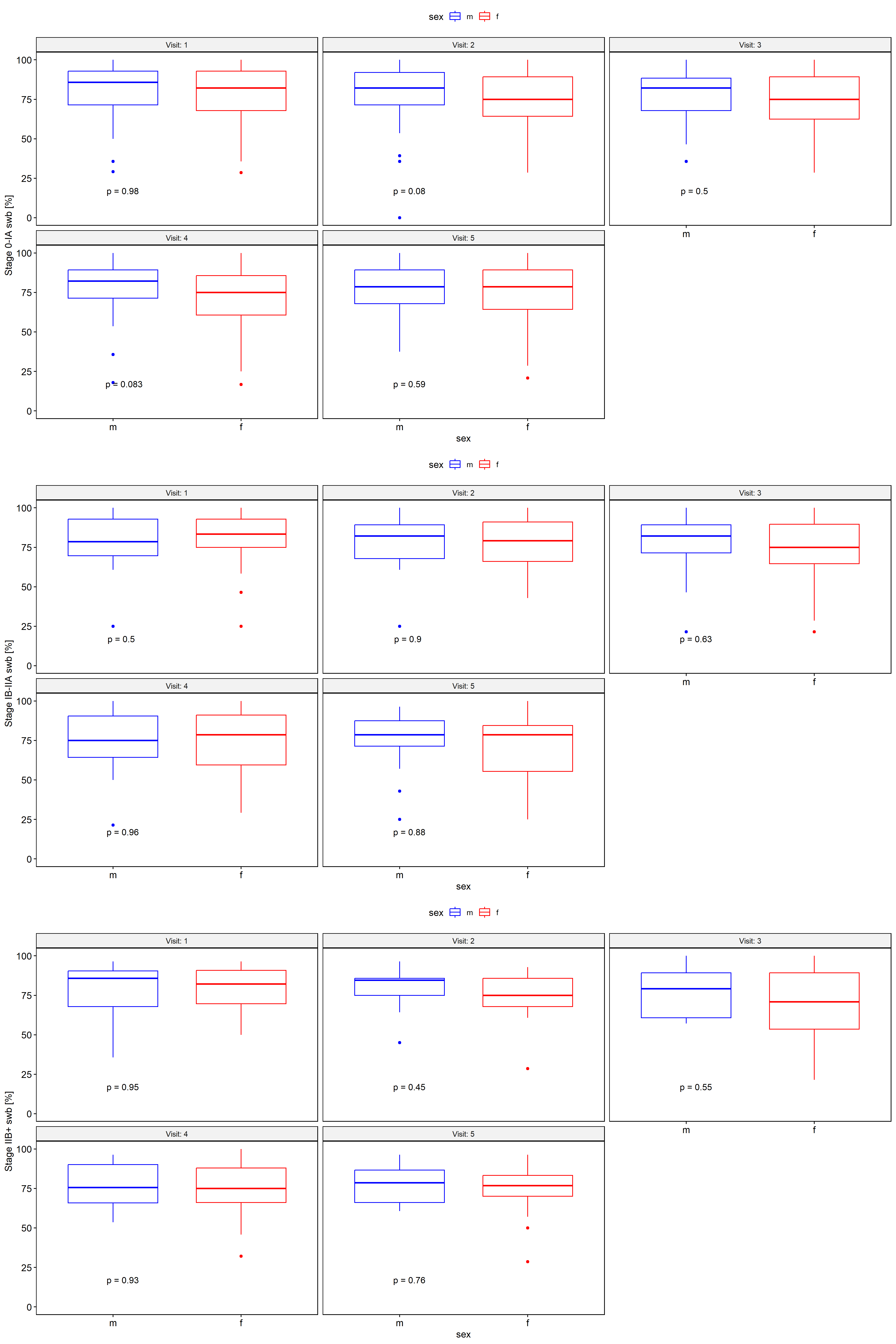


**Fig. 16S** FACT-M Subscale Functional Well-being for men and women, differentiated by tumor stage, shown for each visit


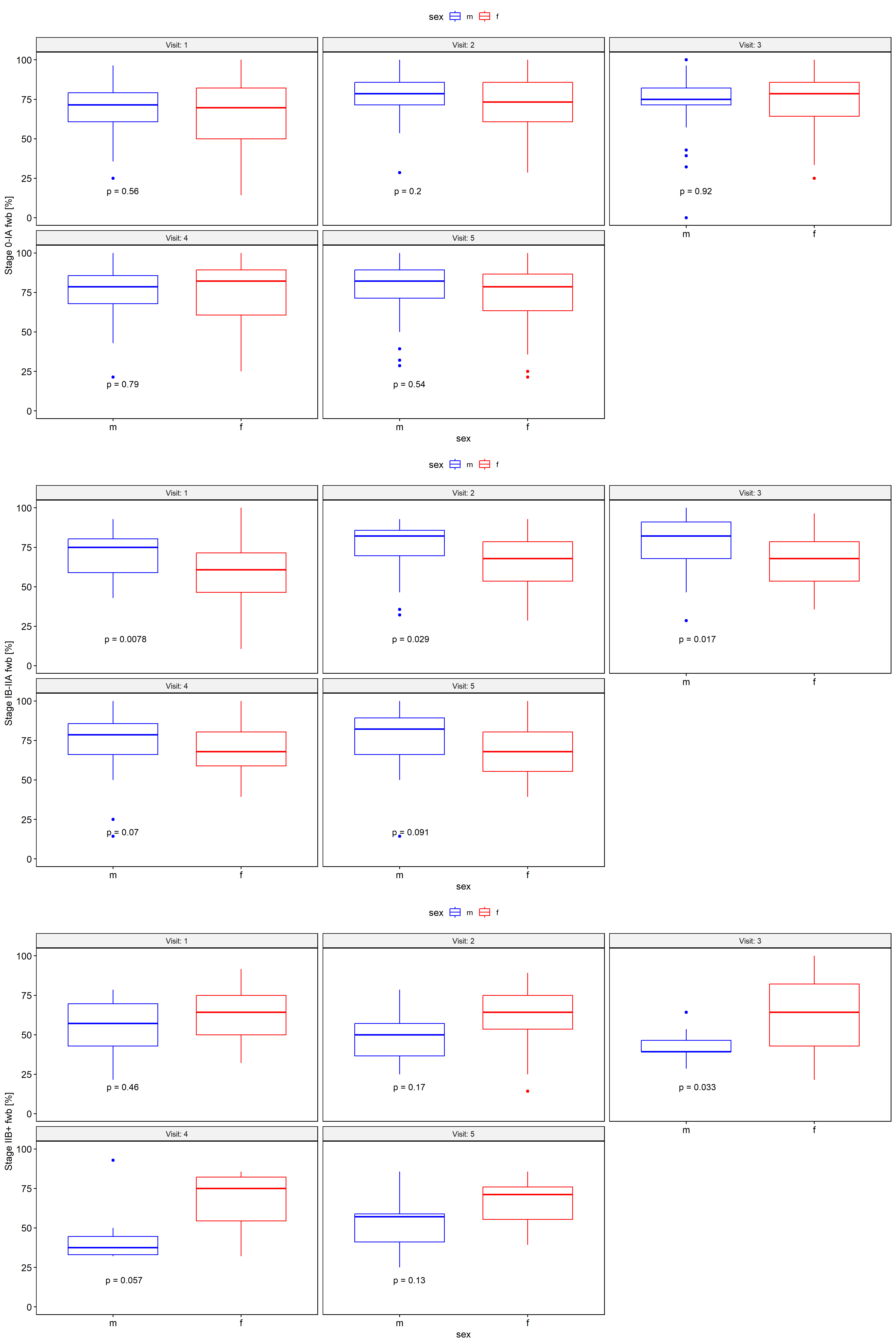


**Fig. 17S** FACT-M Melanoma Subscale for men and women, differentiated by tumor stage, shown for each visit


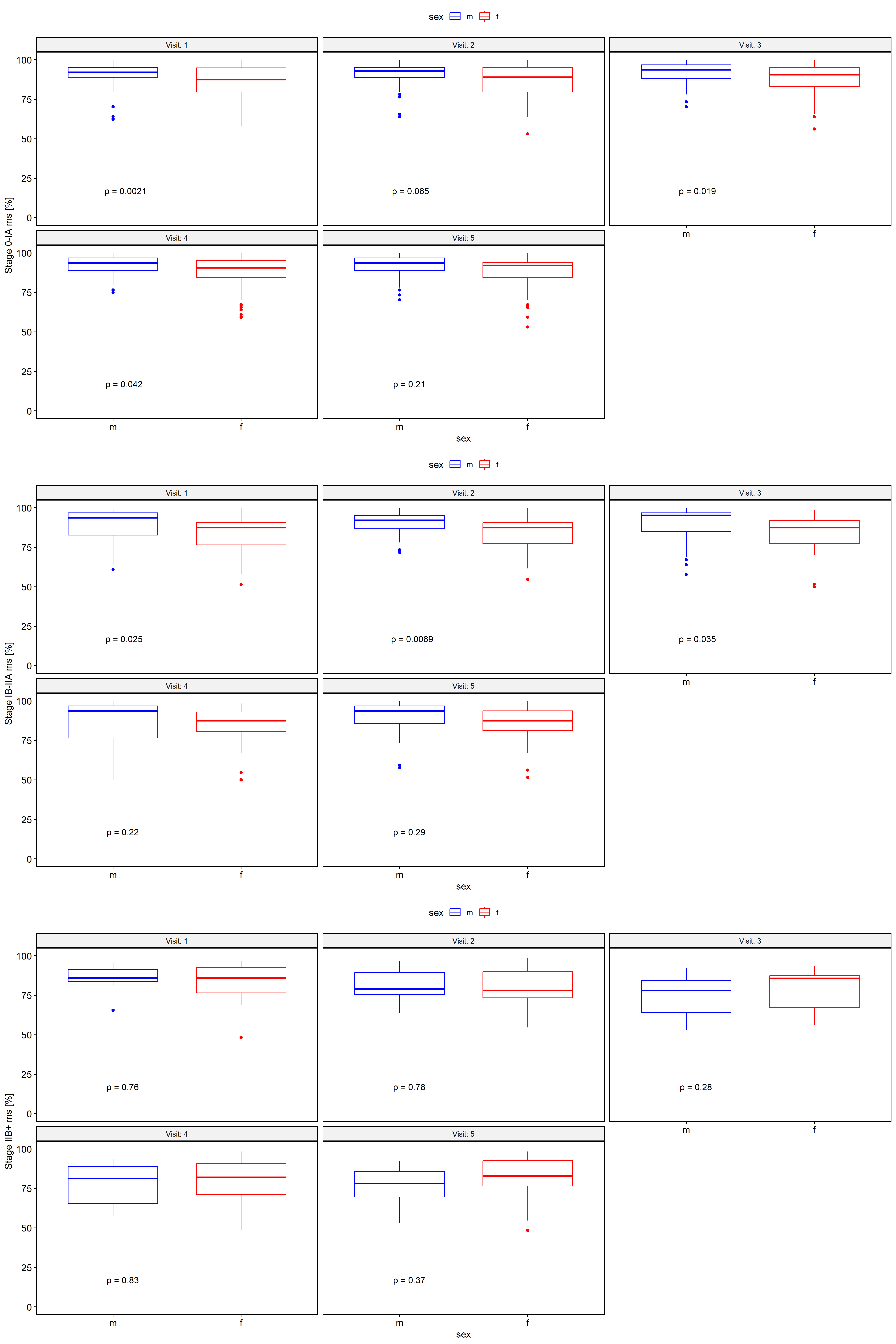


**Fig. 18S** FACT-M Melanoma Surgery Subscale for men and women, differentiated by tumor stage, shown for each visit


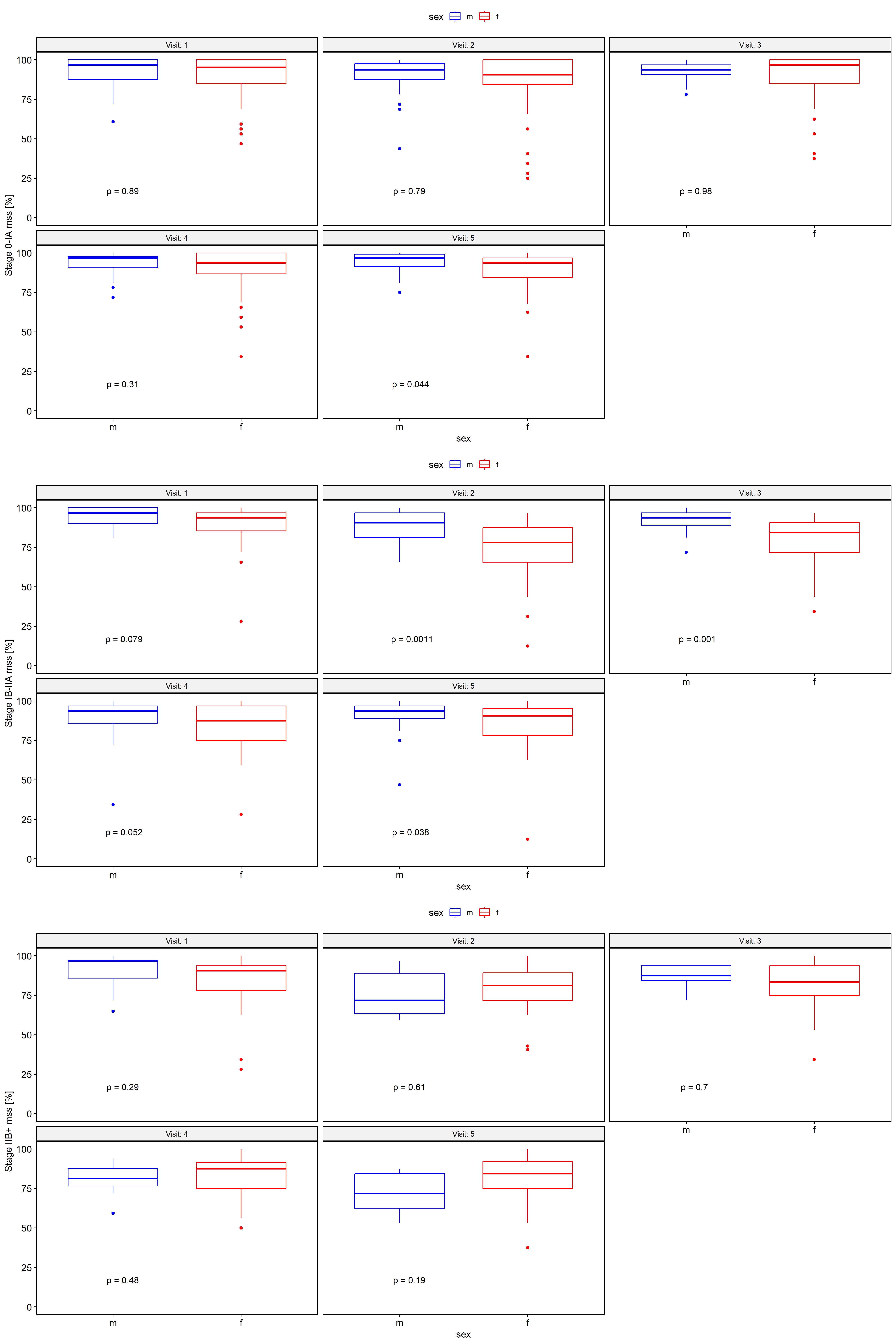


**Fig. 19S** FACT-M for patients without (FALSE)(n=168) and with (TRUE)(n=53) psycho-oncological counseling


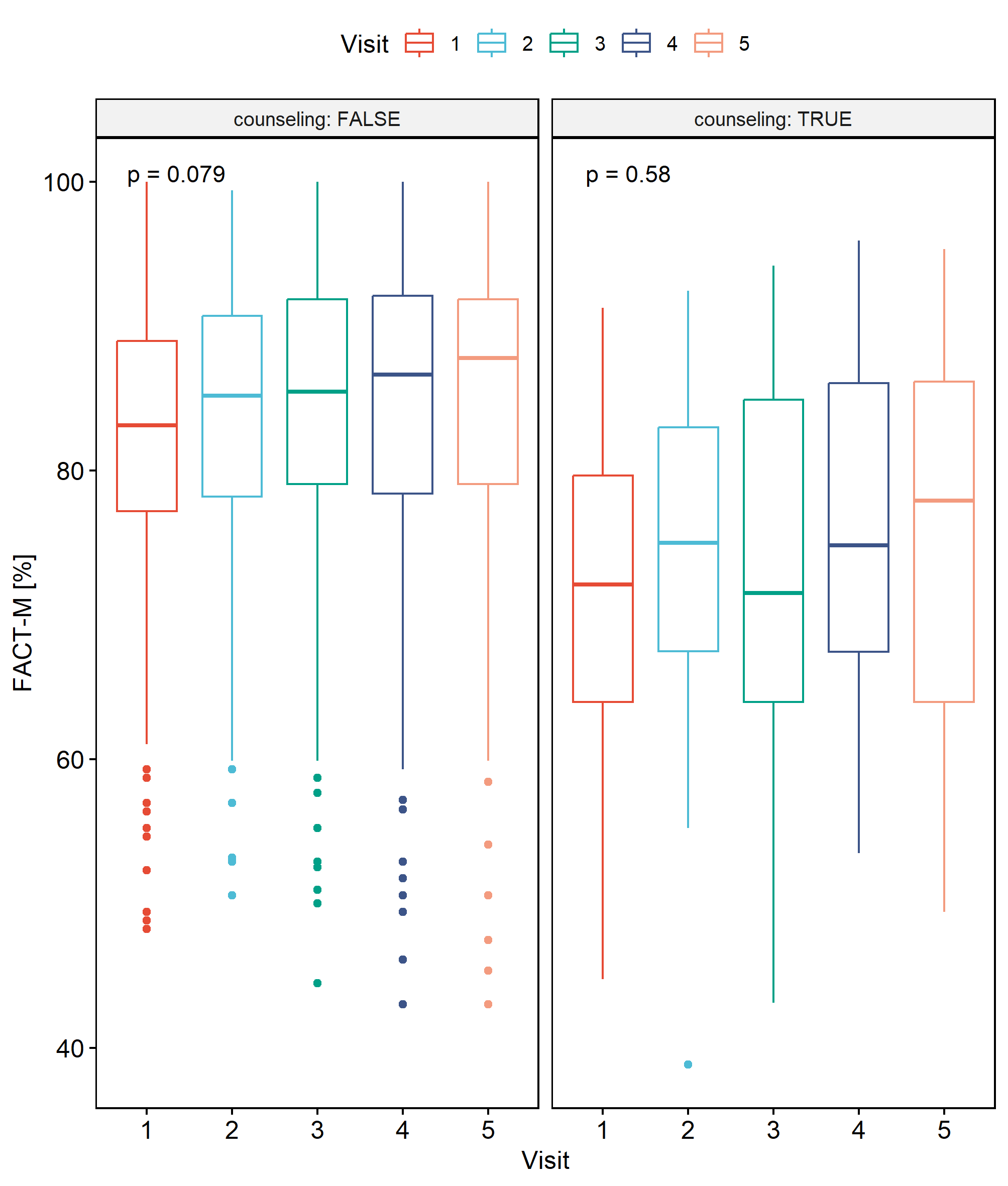


**Fig. 20S** FACT-M for patients without (FALSE)(n=181) and with (TRUE)(n=40) rehabilitation


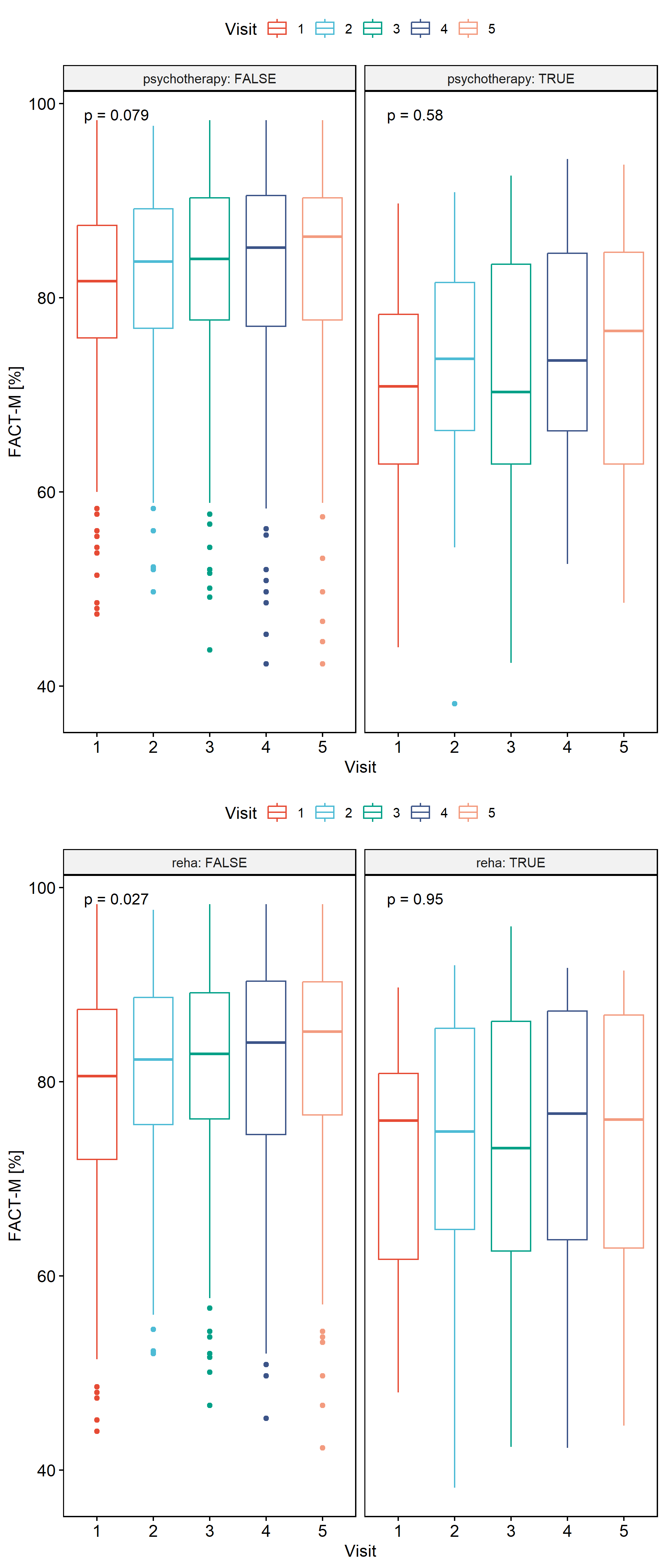


**Fig. 21S** FACT-M and HSI show a strong and significant correlation


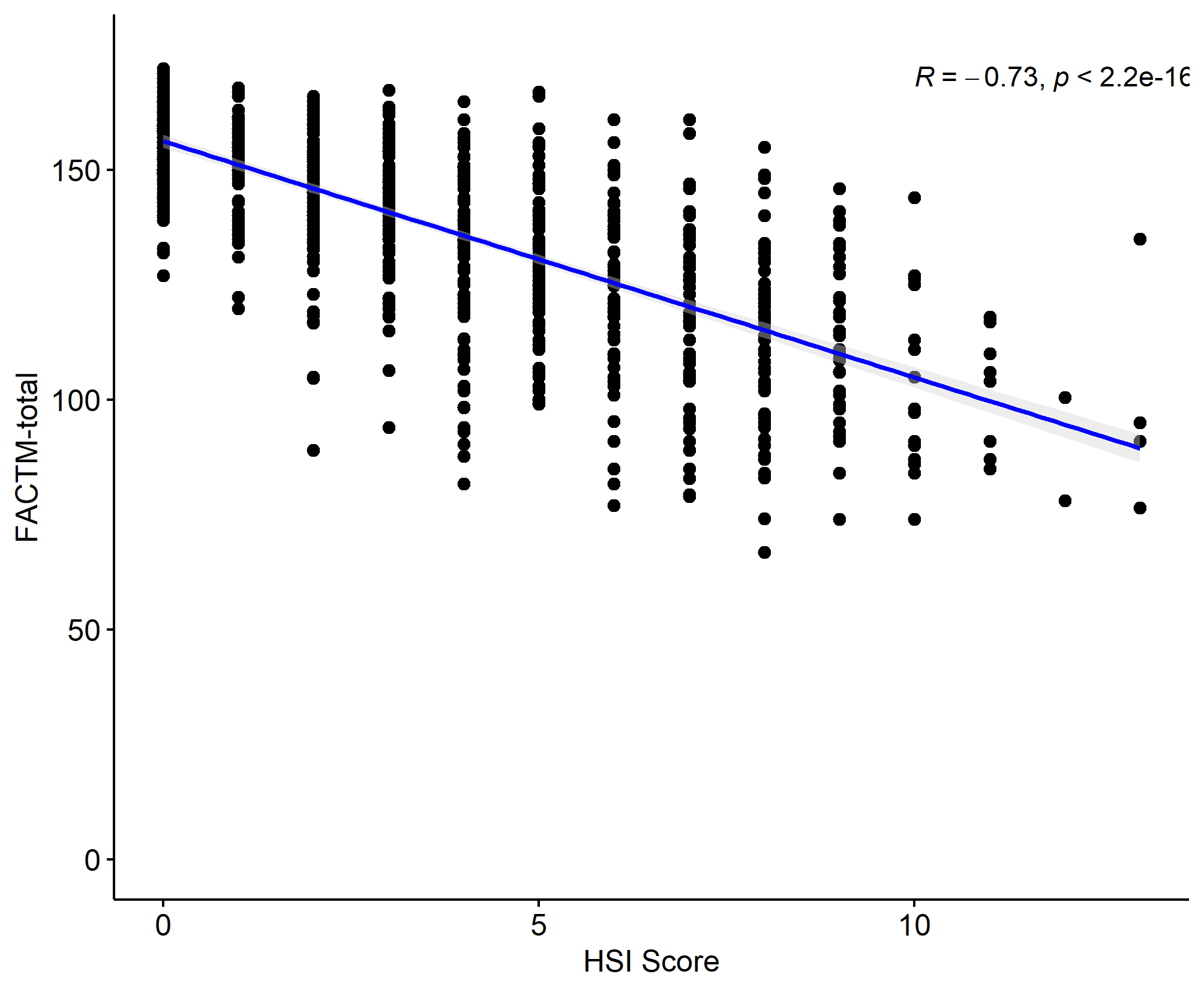


Approximately 53% of the variance of FACT-M can be explained by HSI score.

**Fig. 22S** FACT-M and WHO-5 show a strong and significant correlation


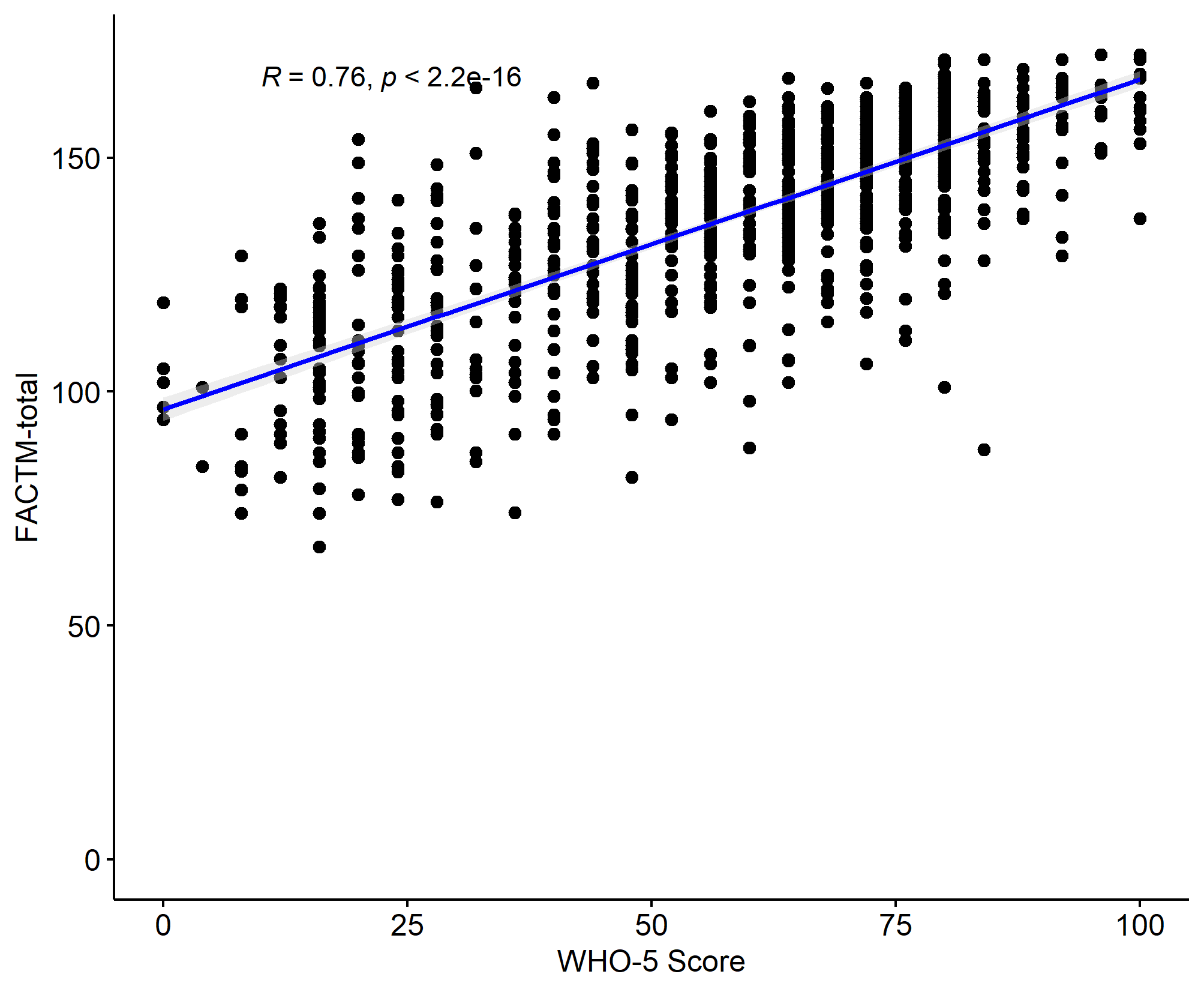


Approximately 58% of the variance of FACT-M can be explained by WHO-5 score.

**Sample size estimation**

Sample size was estimated for a single primary endpoint (HRQoL) with the following assumptions: power 0.80; significance level 0.05; Cohen's d=0.4; two-sided testing.
This resulted in 99 patients in each group, i.e. 198 patients in total.

Under the assumption of a drop-out rate of 10%, 220 patients were planned.

In addition, we performed a power analysis for subgroups. In the last visit (supplement table 4S) 20 patients were in stage II and 23 in stage III. Based on Cohen's d=0.8 (large effect), alpha=0.05 a power of 82% (one-sided test) is achieved.

**Efforts to address potential sources of bias**

To minimize bias, all melanoma patients visiting the clinic in the time period were invited to participate; patients were contacted by E-Mail to answer follow-up questions; the electronic data collection tool had mandatory data items to minimize missing data.

**Fig. 23S** STROBE Flow chart

Drop out in follow up (FU) (n=28)
- Lost to FU (n=11)
- Technical problems (n=3)
- Refuse to participate (n=14)

Completed FU for 12 months
(n=193)

Analyzed (n=221)

Included in the study, according to study criteria
(n=221)

Excluded (n=8)
- < 18 (n=1)
- Unavailable data (n=2)
- Refuse to participate (n=5)

Assessed for eligibility
from 2016-04-20 to 2018-07-09
(n=229)

**Fig.24S** FACT-M total score (a) and subscale emotional well-being (b) at first and last visit in relation to tumor thickness

(a)


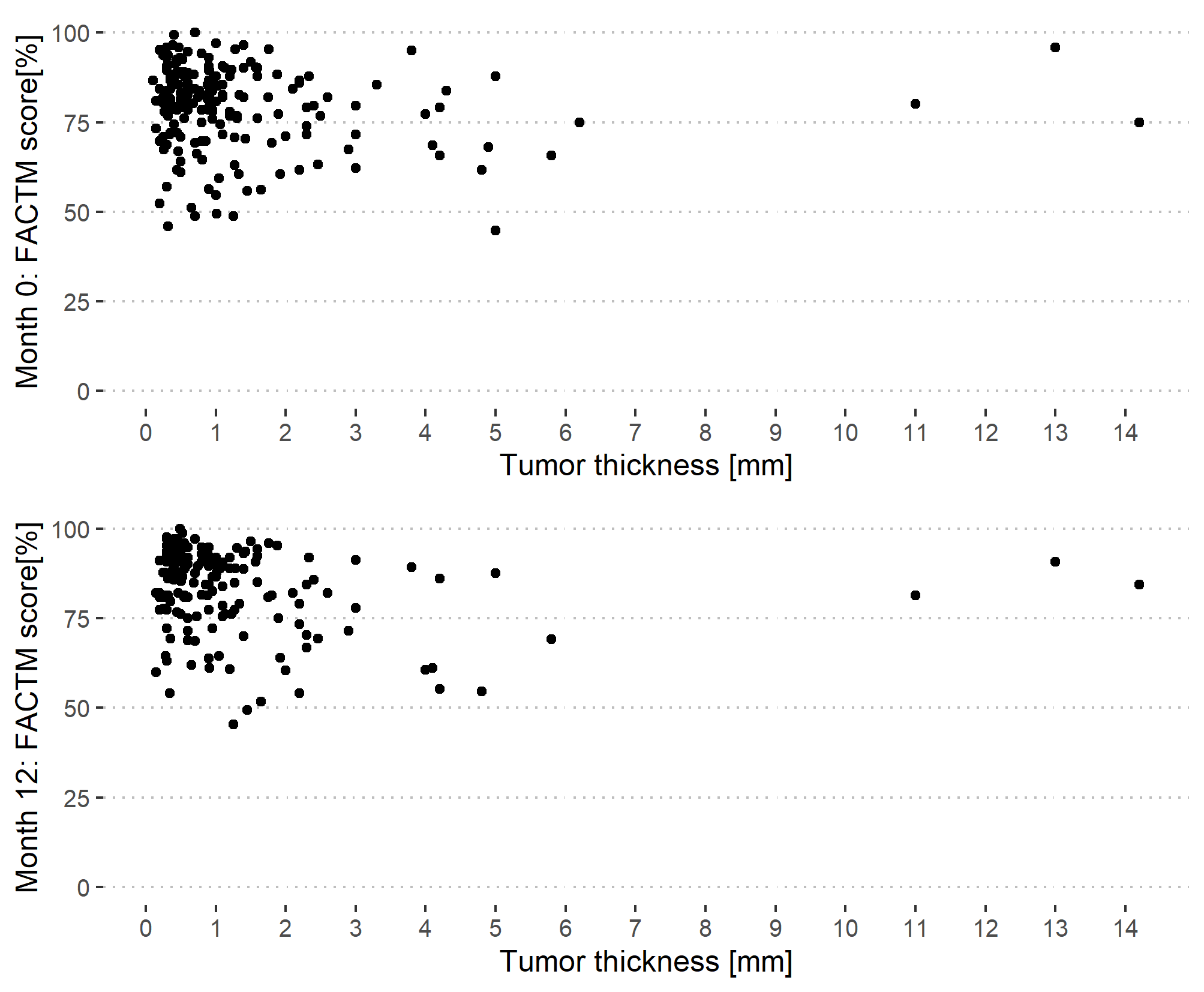


(b)


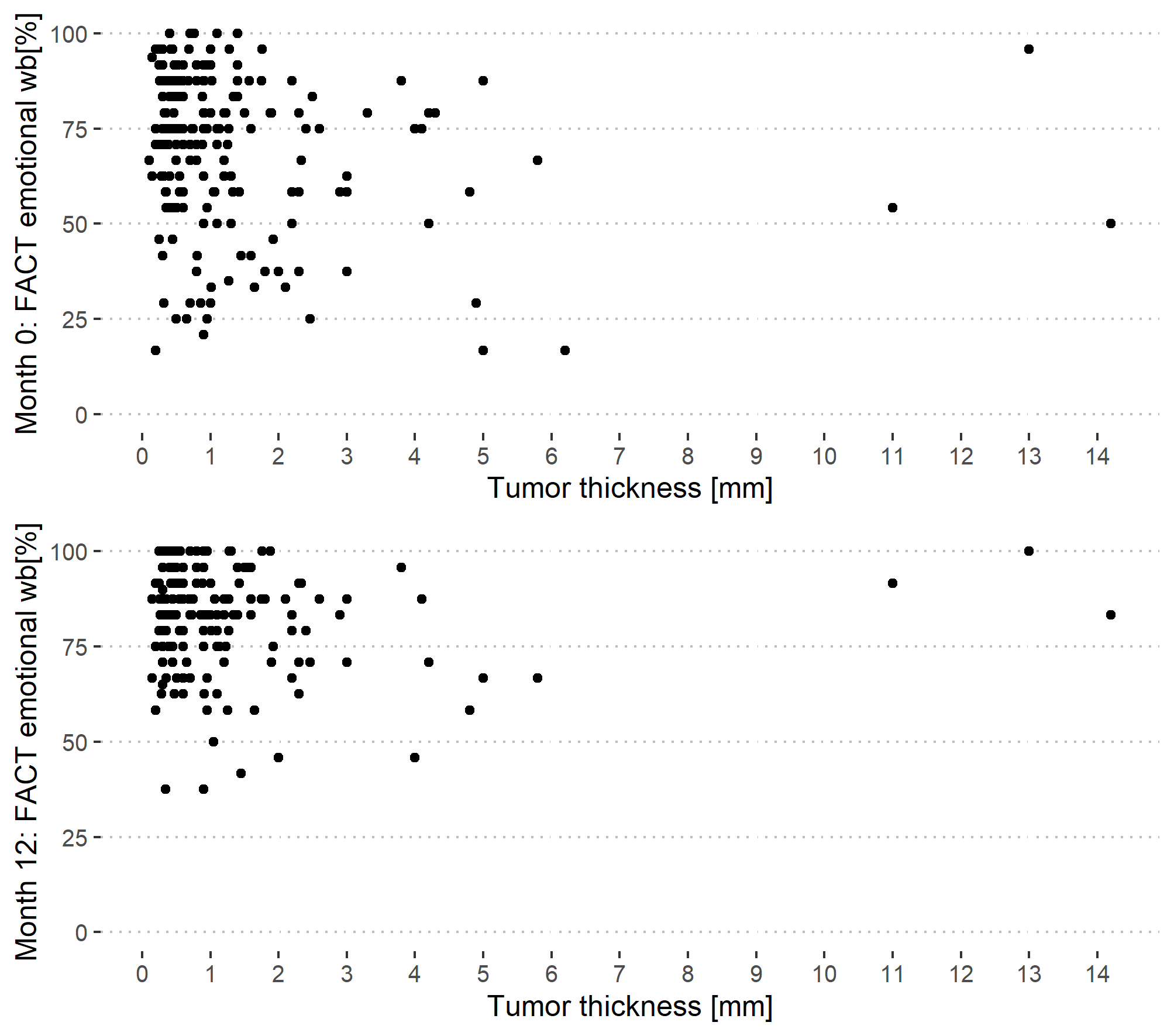


**Full questionnaire (translated from German)**

**You are:**

☐ Single

☐ Married / In a committed relationship

☐ Divorced / Separated

☐ Widowed

**Does your family have a history of cancer / tumors?**

☐ Yes

☐ No

If Yes:
☐ Melanoma

☐ Other / unknown

**What is your level of education?**

□ Still at school

□ No school leaving certificate

□ Primary/secondary school certificate/ 8th grade Polytechnic high school

□ Secondary education/ intermediate education/ 10th grade Polytechnic high school

□ Technical baccalaureate (entrance qualification for universities of applied sciences)

□ Baccalaureate / higher education entrance qualification/ Extended secondary school

□ Completed university degree

□ Other school leaving qualification

**Current diseases**

In the following list, mark your current diseases or injuries. Also indicate whether a physician has diagnosed ore treated these diseases.

|  | Yes, own opinion | Yes, physician´s diagnosis | No |
| --- | --- | --- | --- |
| 01 Injury due to an accident (e.g. in back or limbs, or burns) | □ | □ | □ |
| 02 Musculoskeletal disease in back, limbs or other part of the body (e.g. repeated pain in joint or muscle, sciatica, rheumatism, arthritis) | □ | □ | □ |
| 03 Cardiovascular disease (e.g. hypertension, coronary heart disease) | □ | □ | □ |
| 04 Respiratory disease (e.g. repeated infections of the respiratory tract, bronchial asthma, emphysema) | □ | □ | □ |
| 05 Mental disorder (e.g. depression, “burn-out”, anxiety or insomnia) | □ | □ | □ |
| 06 Neurological or sensory disease (e.g. hearing or visual disease, migraine, epilepsy) | □ | □ | □ |
| 07 Digestive disease / condition (e.g. gastritis, gall stones, liver or pancreatic disease, repeated constipation) | □ | □ | □ |
| 08 Genitourinary disease (e.g. infection in urinary tract, gynecological disease or prostate) | □ | □ | □ |
| 09 Skin disease (e.g. allergic or other rash, varicose veins) | □ | □ | □ |
| 10 Endocrine or metabolic disease (e.g. diabetes, severe obesity or gout) | □ | □ | □ |
| 11 Blood disease (e.g. anemia, other bloed disorder or defect | □ | □ | □ |
| 12 Birth defects | □ | □ | □ |
| 13 Other disorder or disease | □ | □ | □ |


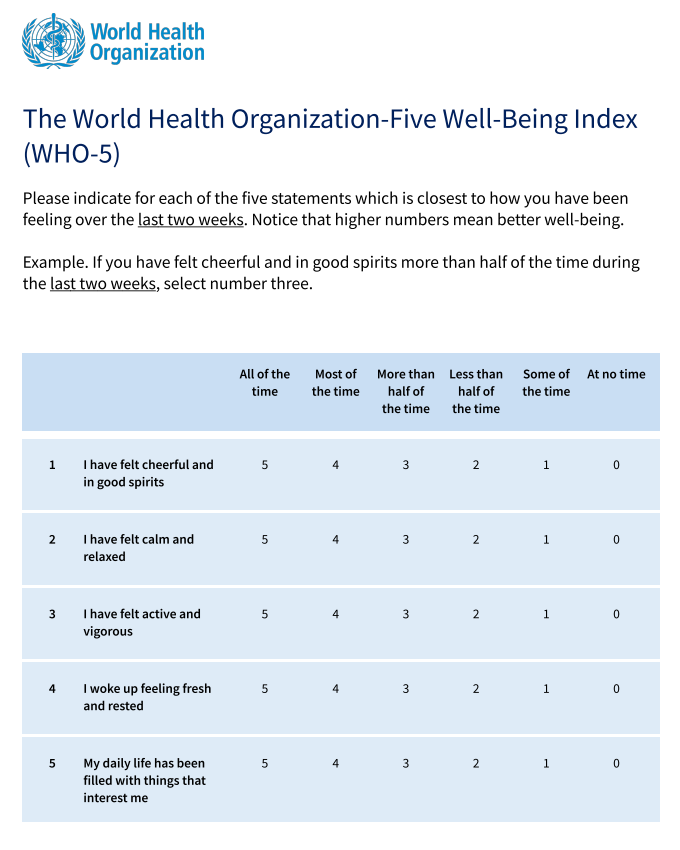
**WHO-5**

**Hornheider Screening Instrument (HSI)**

How have you felt physically in the last three days?

☐ Rather good – 0

☐ Moderate – 1

☐ Rather poor – 2

How have you felt emotionally in the last three days?

☐ Rather good – 0

☐ Moderate – 1

☐ Rather poor – 2

Is there something, unrelated to your current illness, that burdens you greatly?

☐ Yes – 2

☐ No – 0

Do you have someone to talk to about your worries and fears?

☐ Yes – 0

☐ No – 2

Is anyone in your family particularly burdened by your hospital stay?

☐ Yes – 2

☐ No – 0

Are you able to relax internally during the day?

☐ Yes – 0

☐ No – 2

How well do you feel informed about your illness and treatment?

☐ Rather well – 0

☐ Moderate – 1

☐ Rather poorly – 2

Note: The Hornheide Screening Instrument (HSI) is a validated German tool for identifying cancer patients in need of psycho-oncological support. The above items represent an English translation adapted for explanatory purposes; the validated version is available in German.

**FACT-M**

FACT-M is a licensed questionnaire. It is available from:

https://www.facit.org/measures/fact-m
